# Neuronal Dystroglycan regulates postnatal development of CCK/cannabinoid receptor-1 interneurons

**DOI:** 10.1186/s13064-021-00153-1

**Published:** 2021-08-06

**Authors:** Daniel S. Miller, Kevin M. Wright

**Affiliations:** 1 Neuroscience Graduate Program, Oregon Health & Science University, Portland, OR 97239 USA; 2 Vollum Institute, Oregon Health & Science University, VIB 3435A, 3181 SW Sam Jackson Park Road, L474, Portland, OR 97239-3098 USA

**Keywords:** Dystroglycan, Cannabinoid receptor, Cholecystokinin, Interneuron, Synapse, Apoptosis

## Abstract

**Background:**

The development of functional neural circuits requires the precise formation of synaptic connections between diverse neuronal populations. The molecular pathways that allow GABAergic interneuron subtypes in the mammalian brain to initially recognize their postsynaptic partners remain largely unknown. The transmembrane glycoprotein Dystroglycan is localized to inhibitory synapses in pyramidal neurons, where it is required for the proper function of CCK+ interneurons. However, the precise temporal requirement for Dystroglycan during inhibitory synapse development has not been examined.

**Methods:**

In this study, we use *NEX*^*Cre*^ or *Camk2a*^*CreERT2*^ to conditionally delete *Dystroglycan* from newly-born or adult pyramidal neurons, respectively. We then analyze forebrain development from postnatal day 3 through adulthood, with a particular focus on CCK+ interneurons.

**Results:**

In the absence of postsynaptic Dystroglycan in developing pyramidal neurons, presynaptic CCK+ interneurons fail to elaborate their axons and largely disappear from the cortex, hippocampus, amygdala, and olfactory bulb during the first two postnatal weeks. Other interneuron subtypes are unaffected, indicating that CCK+ interneurons are unique in their requirement for postsynaptic Dystroglycan. Dystroglycan does not appear to be required in adult pyramidal neurons to maintain CCK+ interneurons. *Bax* deletion did not rescue CCK+ interneurons in *Dystroglycan* mutants during development, suggesting that they are not eliminated by canonical apoptosis. Rather, we observed increased innervation of the striatum, suggesting that the few remaining CCK+ interneurons re-directed their axons to neighboring areas where *Dystroglycan* expression remained intact.

**Conclusion:**

Together these findings show that Dystroglycan functions as part of a synaptic partner recognition complex that is required early for CCK+ interneuron development in the forebrain.

**Supplementary Information:**

The online version contains supplementary material available at 10.1186/s13064-021-00153-1.

## Background

Proper function of neural circuits requires precise connections between specific populations of excitatory pyramidal and inhibitory neurons. GABAergic interneurons are a highly diverse group of neurons that control brain function by synchronizing and shaping the activity of populations of excitatory pyramidal neurons (PyNs) [1–5]. In mice and humans, the majority of interneurons in the cortex and hippocampus are produced in the medial and caudal ganglionic eminences (MGE and CGE) of the ventral forebrain, and migrate long distances to their final destinations [6–8]. The importance of interneurons for brain function is underscored by their involvement in a wide variety of neurodevelopmental and neurological disorders including autism, schizophrenia, seizures, and Alzheimer’s disease [9–12].

The proper integration of inhibitory interneurons into neural circuits during development relies on multiple processes such as proliferation, migration, axon guidance, cell death, synaptic target selection, synapse formation (synaptogenesis) and synaptic maintenance. Although much progress has been made in identifying candidate molecules that regulate inhibitory synaptogenesis, our understanding of how molecularly defined subtypes of inhibitory interneurons initially identify specific postsynaptic target cells is lacking [13, 14]. One prominent hypothesis for explaining how diverse interneuron subtypes recognize one another during synapse development is the “molecular code” hypothesis, whereby different cell types use unique pairs or complexes of cell adhesion molecules to select their target cells [15–18]. Cell adhesion molecules are ideally suited to regulate synaptic target recognition due to their large diversity and presence at pre- and postsynaptic membranes. Several recent studies support the idea that cell adhesion molecules are key players in regulating subcellular targeting and synaptic specificity [19–22]. Although many families of cell adhesion molecules have been implicated in controlling synapse development, they are often involved in multiple aspects of neural circuit development, making it difficult to determine their precise role in mediating synaptic specificity.

**Fig. 1:**
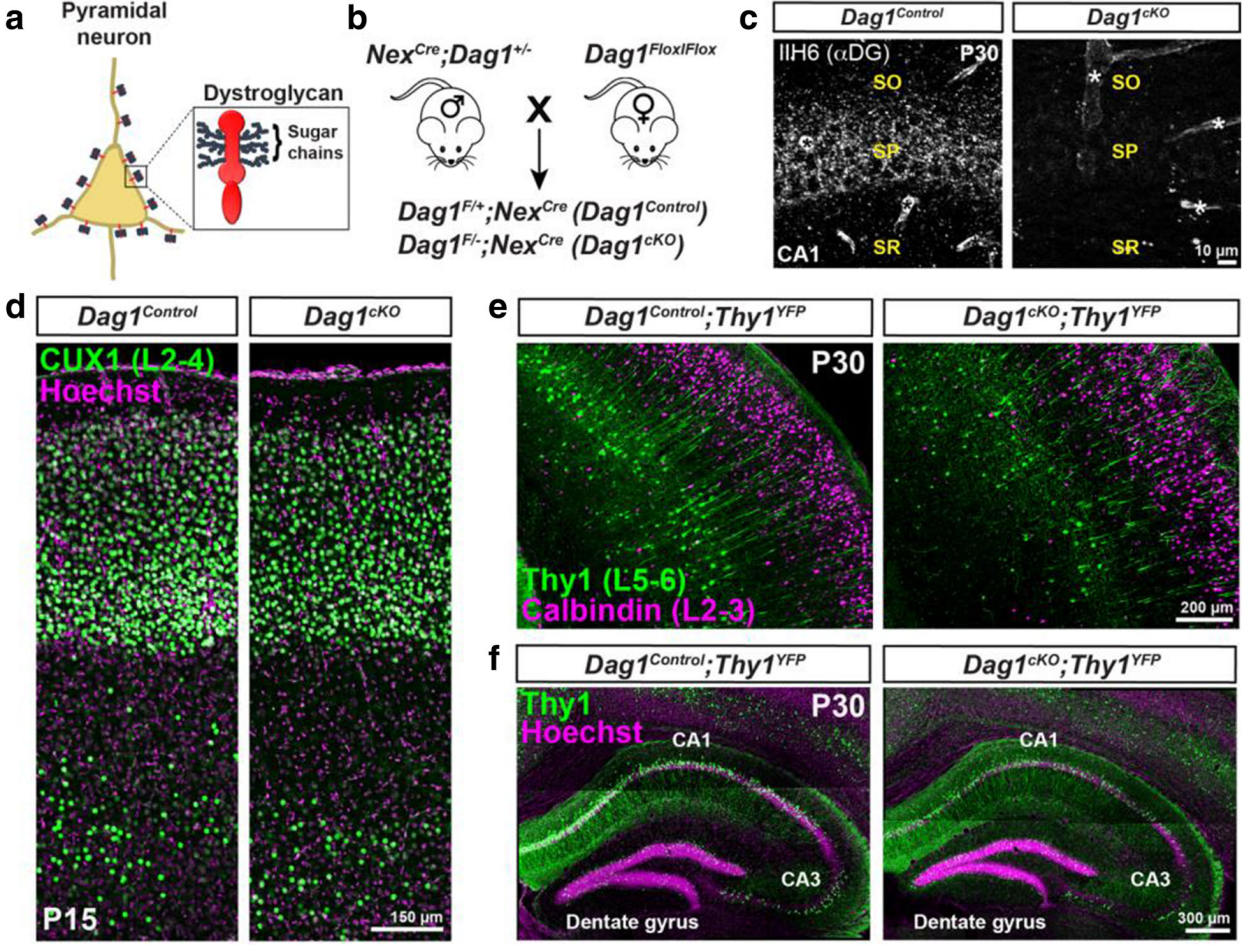
Neuronal *Dystroglycan* is not required for pyramidal neuron migration. (**a**) Schematic of Dystroglycan on pyramidal neurons. Inset shows the structure of Dystroglycan and sugar chain moieties present on the extracellular subunit. (**b**) Mouse breeding scheme for generating pyramidal neuron-specific *Dag1* conditional knockout mice using *Nex*^*Cre*^ driver mice. (**c**) Immunostaining for Dystroglycan in the hippocampal CA1 region of P30 *Dag1*^*Control*^ mice (left panel) shows punctate Dystroglycan protein on the soma and proximal dendrites of pyramidal neurons, whereas *Dag1*^*cKO*^ mice (right panel) lack perisomatic staining. Asterisks denote Dystroglycan staining on blood vessels which is retained in *Dag1*^*cKO*^ mice. (**d**) Coronal sections from P15 *Dag1*^*Control*^ and *Dag1*^*cKO*^ cortex were immunostained for upper layer marker CUX1 (L2–4). (**e**) Coronal sections of the cortex from P30 *Dag1*^*Control*^ and *Dag1*^*cKO*^ mice crossed with a *Thy1*^*YFP*^ reporter mouse to sparsely label layer 5–6 pyramidal neurons (green) and stained for Calbindin (magenta) to label layer 2–3 pyramidal neurons. (**f**) Coronal sections of the hippocampus from P30 *Dag1*^*Control*^ and *Dag1*^*cKO*^ mice crossed with a *Thy1*^*YFP*^ reporter mouse to label excitatory neurons (green) in the CA regions and dentate gyrus

Dystroglycan is a cell adhesion molecule widely expressed throughout the body including the developing and adult brain. Dystroglycan is extensively glycosylated, and mutations in at least 19 genes that participate in synthesizing and elongating specific O-mannose sugar chains on Dystroglycan result in a form of congenital muscular dystrophy called dystroglycanopathy, characterized by muscle weakness and neurological defects of varying severity [23–25]. *Dystroglycan* (*Dag1*) is expressed by multiple cell types in the developing nervous system, including neuroepithelial cells, astrocytes, oligodendrocytes, and excitatory neurons [26, 27]. Loss of *Dystroglycan* function in the nervous system phenocopies the most severe forms of dystroglycanopathy, and causes multiple structural brain and retinal abnormalities due to its indirect role in regulating neuronal migration and axon guidance [28–33]. However, some individuals with milder forms of dystroglycanopathy exhibit cognitive impairments even in the absence of detectable brain malformations, suggesting a possible role for *Dystroglycan* at later stages of brain development including synaptogenesis [34, 35]. In PyNs, Dystroglycan is highly concentrated on the cell body and proximal dendrites where it is a major postsynaptic component of inhibitory synapses (Fig. 1A [27, 36–38]). However, because of its importance in early aspects of brain development, the role of Dystroglycan at synapses has remained obscure. Using a mouse genetic approach to selectively delete *Dystroglycan* from PyNs, a recent study showed that *Dystroglycan* is required for the formation and maintenance of CCK+ interneuron (CCK+ IN) synapses in adult animals, but its specific role in the early development of these interneurons has not been examined [39].

In this study, we show that postsynaptic Dystroglycan on PyNs is required for the proper development of presynaptic CCK+ INs throughout the forebrain. In mice lacking *Dystroglycan* in PyNs, CCK+ INs fail to elaborate their axons during the first postnatal week and are largely absent by P10. CCK+ INs were not rescued by genetic deletion of *Bax* suggesting that CCK+ INs may undergo *Bax*-independent cell death or fail to differentiate in the absence of Dystroglycan. Some remaining CCK+ INs retarget their axons into the striatum, where Dystroglycan expression is retained, suggesting that Dystroglycan functions to allow CCK+ INs to recognize their synaptic partners. Collectively, these results demonstrate that Dystroglycan is a critical regulator of CCK+ IN development.

## Results

### CCK+ interneurons are largely absent in mice lacking *Dystroglycan* from pyramidal neurons

To investigate the role of neuronal Dystroglycan in forebrain development, we used a conditional genetic approach to delete *Dystroglycan* selectively from pyramidal neurons (PyNs). We crossed *Dystroglycan* conditional mice (*Dag1*^*Flox/Flox*^) with *Nex*^*Cre*^ driver mice to delete *Dystroglycan* in all postmitotic excitatory neurons of the forebrain except Cajal-Retzius cells, beginning at E12.5 [40–43]. Control (*Nex*^*Cre*^*;Dag1*^*F/+*^) and conditional knockout mice (*Nex*^*Cre*^*;Dag1*^*F/−*^*)* are hereafter referred to as *Dag1*^*Control*^ and *Dag1*^*cKO*^ mice, respectively (Fig. 1B). We verified the recombination specificity of the *Nex*^*Cre*^ line by crossing it with a reporter mouse that expresses mCherry in the nuclei of *Cre*-recombined cells (*R26*^*LSL-H2B-mCherry*^ [44]). mCherry+ nuclei were detected in excitatory neurons of the forebrain including the cortex, hippocampus, amygdala, and nucleus of the lateral olfactory tract (nLOT) (Fig. S1A). Importantly, mCherry+ nuclei did not overlap with markers for interneurons (CB_1_R, PV, Calbindin) or astrocytes (GFAP), confirming the specificity of the *Nex*^*Cre*^ mouse (Fig. S1B, C). In *Dag1*^*Control*^ mice, Dystroglycan staining was observed as puncta concentrated primarily on the cell bodies and proximal dendrites of PyNs, as well as blood vessels (Fig. 1C). In *Dag1*^*cKO*^ mice, Dystroglycan staining was absent from PyNs but was still present on blood vessels, confirming the specificity of the conditional knockout.

Deletion of *Dystroglycan* from neuroepithelial cells results in disrupted neuronal migration, axon guidance, and dendrite development in the brain, spinal cord and retina [28–33]. In contrast, deletion of *Dystroglycan* from PyNs with *Nex*^*Cre*^ did not affect overall brain architecture, consistent with previous results [32]. Cortical lamination in *Dag1*^*cKO*^ mice was normal based on CUX1 immunostaining of layer 2–4 PyNs and labeling of layer 5–6 and hippocampal PyNs with a *Thy1*^*GFP-H*^ transgenic line (Fig. 1D-F). Therefore, neuronal Dystroglycan is not required for PyN migration in the forebrain**.**

**Fig. 2:**
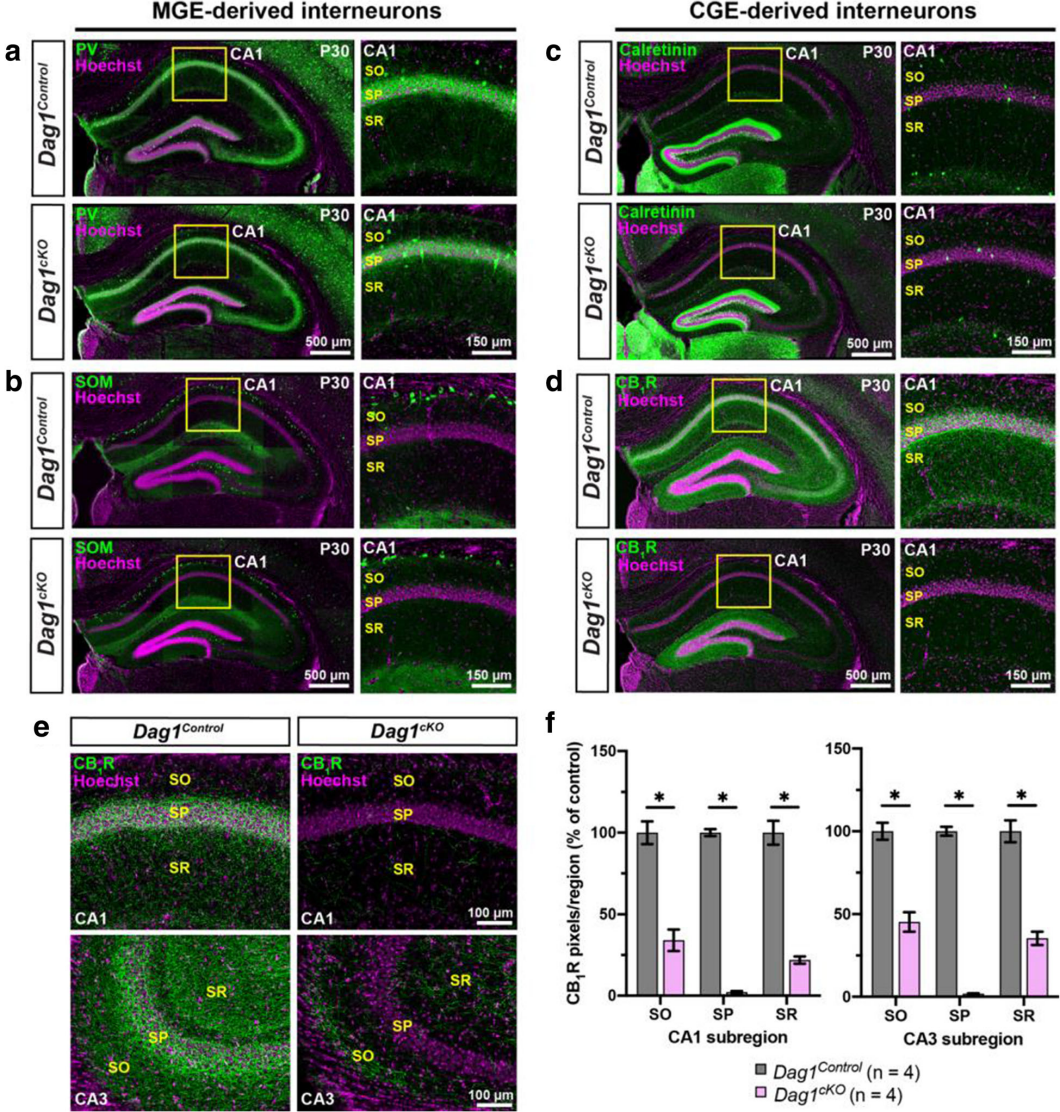
CCK+ interneurons are selectively reduced in mice lacking *Dystroglycan* from pyramidal neurons. (**a-b**) Immunostaining for medial ganglionic eminence (MGE)-derived interneuron markers (green) parvalbumin (PV) (**a**) and somatostatin (SOM) (**b**) show normal innervation of the hippocampus in P30 *Dag1*^*Control*^ and *Dag1*^*cKO*^ mice. Insets (yellow boxed regions) show enlarged images of the CA1. (**c-d**) Immunostaining for caudal ganglionic eminence (CGE)-derived interneuron markers (green) Calretinin (**c**), and CB_1_R (**d**) show normal innervation of Calretinin interneurons in *Dag1*^*Control*^ and *Dag1*^*cKO*^ mice, whereas CB_1_R is largely absent from the CA regions of *Dag1*^*cKO*^ mice. Insets (yellow boxed regions) show enlarged images of the CA1. (**e**) Immunostaining for CB_1_R in hippocampal CA1 (top) and CA3 (bottom) of P30 *Dag1*^*Control*^ and *Dag1*^*cKO*^ mice. (**f**) Quantification of CB_1_R pixels for each CA layer of the CA1 and CA3 shows a significant reduction in CB_1_R staining in *Dag1*^*cKO*^ mice (*P < 0.05, unpaired two-tailed Student’s t-test; *n* = 4 mice/genotype). Data are presented as mean values ± s.e.m. Data are normalized to *Dag1*^*Control*^ signal in each CA layer. CA layers: SO, *stratum oriens*; SP, *stratum pyramidale*; SR, *stratum radiatum*

Forebrain interneurons (INs) are a remarkably diverse population, with multiple molecularly and morphologically distinct IN subtypes forming synapses onto different subcellular domains of PyNs [2, 45, 46]. Since Dystroglycan is localized to inhibitory synapses on the soma and dendrites of PyNs, we examined whether IN development is affected in *Dag1*^*cKO*^ mice. We performed immunostaining with a panel of molecular markers that label IN subpopulations in the hippocampus of adult mice (Fig. 2). In *Dag1*^*Control*^ mice, parvalbumin (PV) and somatostatin (SOM) positive INs, which label the majority of interneurons that originate from the medial ganglionic eminence (MGE), were abundant throughout the hippocampus. The distribution of PV+ and SOM+ cell bodies and their synaptic targeting patterns appeared the same in *Dag1*^*cKO*^ mice, suggesting these populations are unaffected by the loss of *Dystroglycan* (Fig. 2A, B).

We next stained the hippocampus for IN subtypes that originate from the caudal ganglionic eminence (CGE). The distribution and synaptic targeting of Calretinin interneurons, which target other INs as well as PyN dendrites, appeared normal (Fig. 2C [47, 48]). In contrast, we found a dramatic reduction in cannabinoid receptor-1 (CB_1_R) staining in the hippocampus, which labels the axon terminals of cholecystokinin (CCK) + INs (Fig. 2D [49–51]). CB_1_R+ terminals were significantly reduced in all CA subregions (Fig. 2E, F). In both the CA1 and CA3, the magnitude of the reduction varied by layer. CB_1_R+ terminals were most strongly reduced (> 95%) in the *stratum pyramidale* (SP) where CCK+ INs form basket synapses onto PyN cell bodies, and more moderately reduced in the *stratum radiatum* (SR) and *stratum oriens* (SO) where CCK/CB_1_R+ INs synapse onto PyN dendrites (Fig. 2E, F). In contrast, CB_1_R+ terminals were abundant in the dentate gyrus of *Dag1*^*cKO*^ mice (Fig. S2). This is likely because *Nex*^*Cre*^ recombination is restricted to the outer third of granular layer neurons (Fig. S1C [41]).

**Fig. 3:**
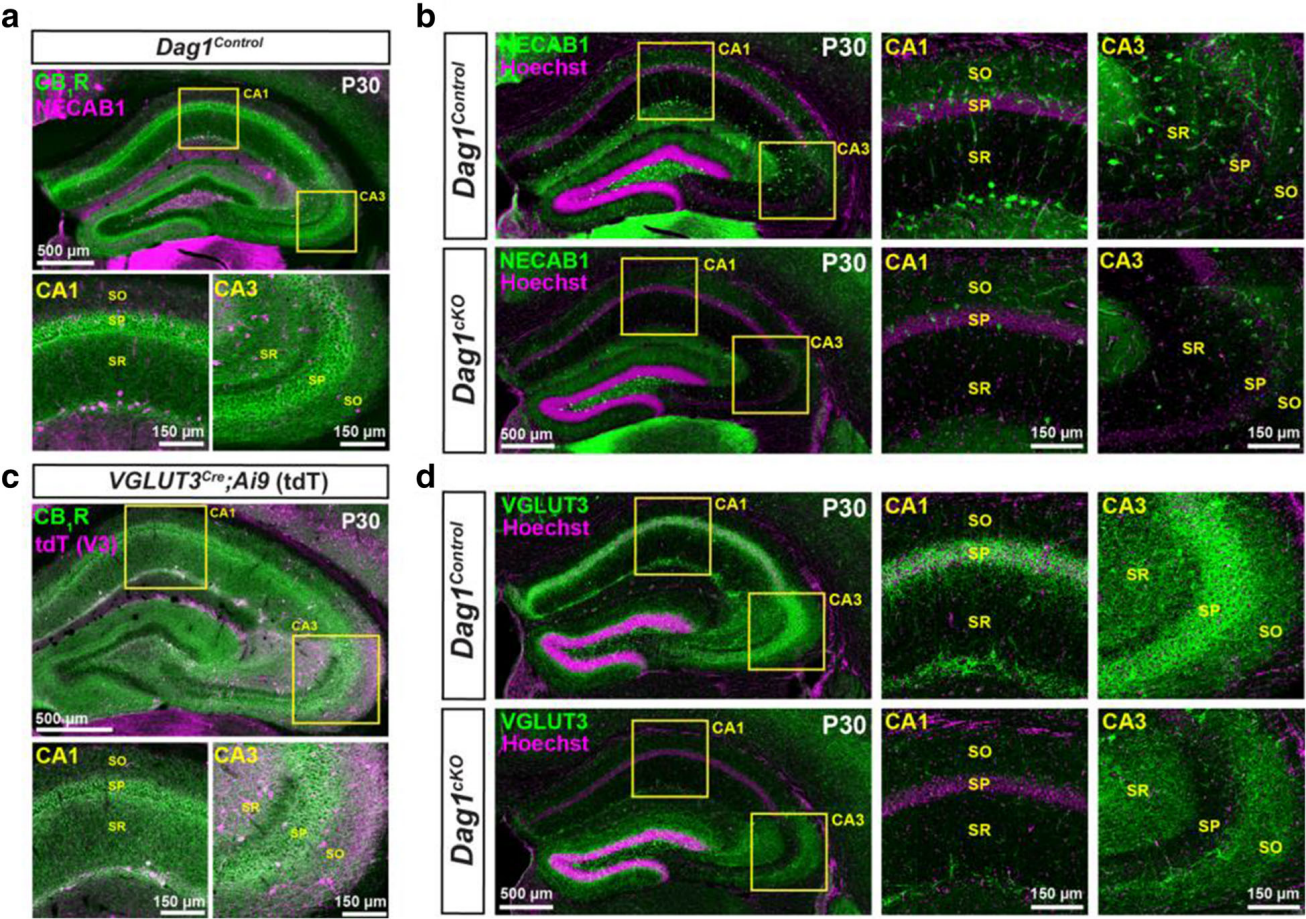
Cell body and synaptic markers for CCK+ interneurons are reduced in *Dag1*^*cKO*^ mice. (**a**) Immunostaining showing the co-localization of CB_1_R (green) and NECAB1 (magenta) in CCK+ interneurons. Insets (yellow boxed regions) show enlarged images of the CA1 and CA3. (**b**) Immunostaining for NECAB1 (green) shows a reduction of NECAB1+ interneurons in the hippocampus of P30 *Dag1*^*cKO*^ mice. Insets (yellow boxed regions) show enlarged images of the CA1 and CA3. (**c**) Immunostaining of hippocampal sections from *VGLUT3*^*Cre*^ mice crossed with a Lox-STOP-Lox-tdTomato (Ai9) reporter mouse showing the co-localization of CB_1_R (green) and VGLUT3 (magenta) in a subset of CCK+ interneurons. Insets (yellow boxed regions) show enlarged images of the CA1 and CA3. (**d**) Immunostaining for VGLUT3 (green) shows a reduction of CCK+ interneuron synaptic terminals in the hippocampus of P30 *Dag1*^*cKO*^ mice. Insets (yellow boxed regions) show enlarged images of the CA1 and CA3. CA layers: SO, *stratum oriens*; SP, *stratum pyramidale*; SR, *stratum radiatum*

The loss of CB_1_R staining in the hippocampus of *Dag1*^*cKO*^ mice could reflect either downregulation of CB_1_R expression or a loss of CCK+ INs. To distinguish between these possibilities, we examined whether other independent markers of CGE-derived CCK+ INs were similarly reduced. These include NECAB1, a calcium binding protein that specifically labels CCK+ IN cell bodies (Fig. 3A) [52], and VGLUT3, a vesicular glutamate transporter enriched at CCK+ IN synapses **(**Fig. 3C**)** [53–55]. Both NECAB1+ cell bodies and VGLUT3+ synaptic terminals were reduced in the hippocampus of *Dag1*^*cKO*^ mice (Fig. 3B, D). Based on the loss of all three markers, we conclude that CCK+ INs are largely absent from the hippocampus of *Dag1*^*cKO*^ mice.

**Fig. 4:**
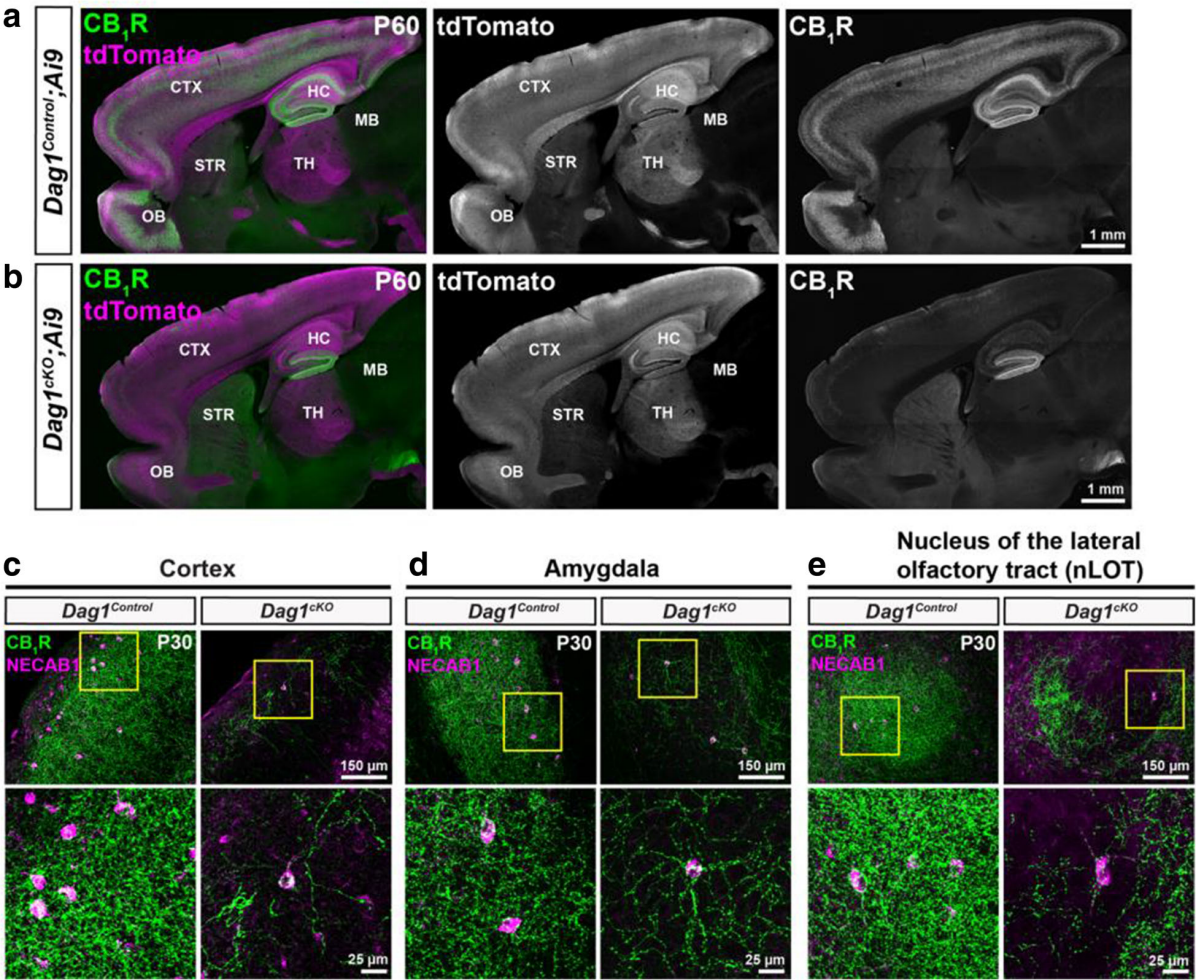
CCK+ interneurons are reduced throughout the forebrain of mice lacking *Dystroglycan* from pyramidal neurons. (**a-b**) Sagittal sections from P60 *Dag1*^*Control*^*;Ai9* (**a**) and *Dag1*^*cKO*^*;Ai9* mice (**b**) immunostained for CB_1_R (green; right panels) and tdTomato/Ai9 (magenta; middle panels). In *Dag1*^*cKO*^*;Ai9* mice, CB_1_R staining is lacking in all the forebrain regions where *Nex*^*Cre*^ drives recombination in excitatory neurons (tdTomato expression, middle panels) including the cortex (CTX), hippocampus (HC), and olfactory bulb (OB). Note the absence of tdTomato signal in the striatum (STR) and midbrain (MB), which are not targeted by *Nex*^*Cre*^. (**c-e**) Immunostaining for CB_1_R (green) and NECAB1 (magenta) in the cortex (**c**), amygdala (**d**), and nucleus of the lateral olfactory tract (**e**) shows the reduction of CCK+ interneuron markers in the forebrain of P30 *Dag1*^*cKO*^ mice (right panels). Enlarged images (yellow boxed regions) show individual NECAB1+ cell bodies (magenta) co-localized with CB_1_R (green)

In addition to the hippocampus, Dystroglycan is present in PyNs of the cortex, amygdala, and nucleus of the lateral olfactory tract (nLOT) [27], which all receive extensive innervation from CCK+ INs [56–58]. Therefore, we assessed whether deletion of *Dystroglycan* from PyNs affects CCK+ INs and their terminals in these forebrain regions. We first performed immunostaining for CB_1_R on sagittal sections from *Dag1*^*Control*^ and *Dag1*^*cKO*^ mice. CB_1_R terminals were largely absent throughout the entire forebrain of *Dag1*^*cKO*^ mice (Fig. 4A, B). Next, we stained P30 *Dag1*^*Control*^ and *Dag1*^*cKO*^ mice for NECAB1 and CB_1_R to label the cell bodies and terminals of CCK+ INs, respectively (Fig. 4C-E). In *Dag1*^*Control*^ mice, NECAB1+ cell bodies were numerous and CB_1_R innervation was extensive in the cortex, amygdala, and nLOT. In contrast, NECAB1+ cell bodies were dramatically reduced, and CB_1_R staining was almost completely absent in all regions of *Dag1*^*cKO*^ mice (Fig. 4C-E). In each region, a few NECAB1+ cell bodies remained in *Dag1*^*cKO*^ mice, and these co-localized with CB_1_R. Therefore, Dystroglycan expressed in PyNs is required broadly in the developing forebrain for the proper integration of CCK+ INs.

### Postnatal development of CCK+ interneurons is impaired in the forebrains of *Dag1*^*cKO*^ mice

**Fig. 5:**
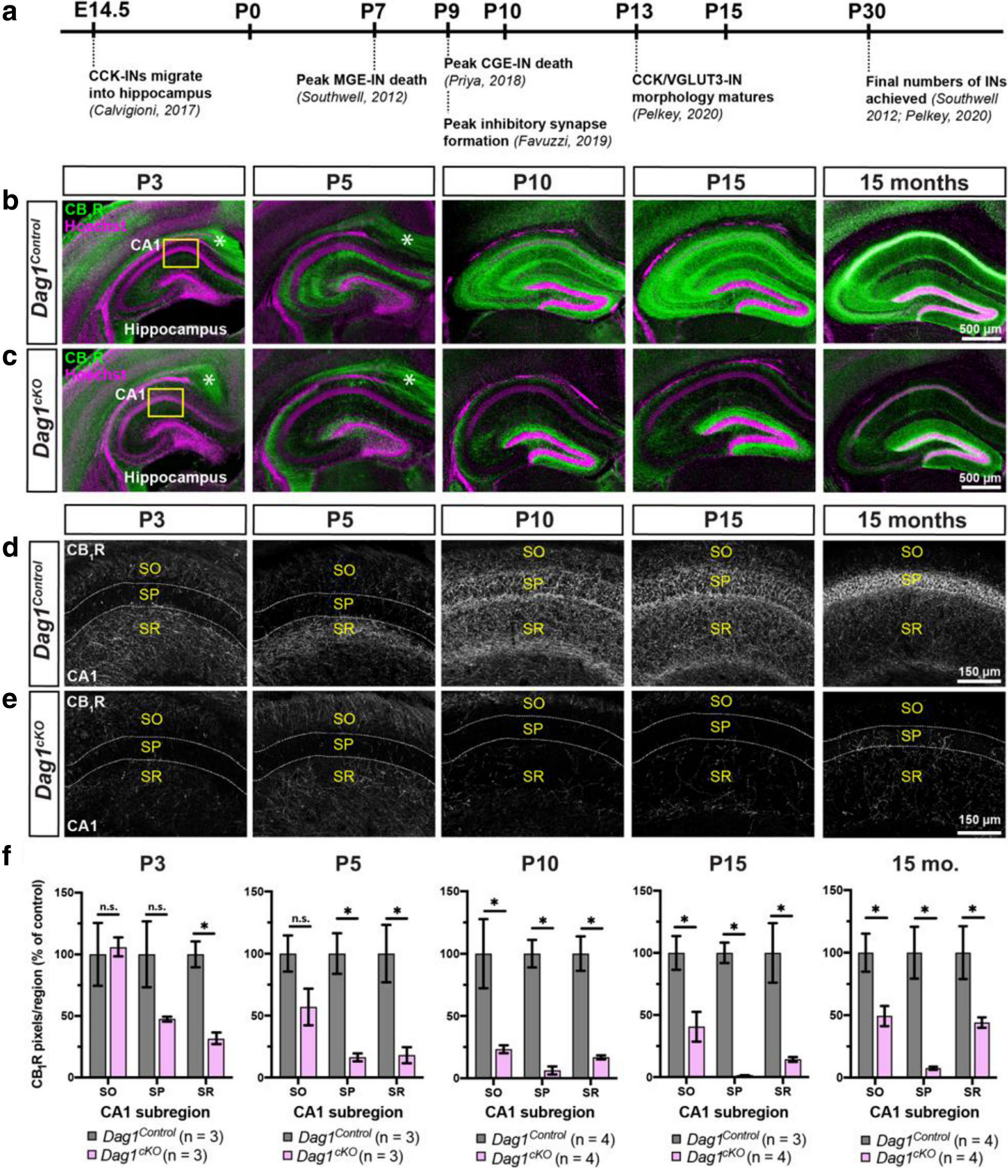
Postnatal development of CCK+ interneurons is impaired in the hippocampus of *Dag1*^*cKO*^ mice. (**a**) Timeline of interneuron developmental milestones including interneuron migration, cell death, and inhibitory synapse formation. (**b-c**) Immunostaining for CB_1_R (green) in the hippocampus of *Dag1*^*Control*^ mice (**b**) shows a progressive increase in CCK+ interneuron axon terminals from P3-P15. In contrast, CB_1_R+ axon terminals are diminished at all ages in *Dag1*^*cKO*^ mice (**c**). Asterisks (P3 and P5) denote the presence of CB_1_R immunoreactivity in pyramidal neuron axons at early postnatal ages. Yellow boxes (**b, c**) indicate approximate locations of high magnification images in (**d-e**)**.** High magnification (20X), single channel images (gray) of CB_1_R+ axon terminals in the CA1 of *Dag1*^*Control*^ (**d**) and *Dag1*^*cKO*^ mice (**e**) from P3–15 months. Dotted white lines indicate the position of the pyramidal cell layer (SP). SO, *stratum oriens*; SP, *stratum pyramidale*; SR, *stratum radiatum*. **f** Quantification of CB_1_R pixels in hippocampal CA1 layers from *Dag1*^*Control*^ (gray) and *Dag1*^*cKO*^ (pink) mice shows significantly reduced CB_1_R staining at all ages examined (*P < 0.05, unpaired two-tailed Student’s t-test; *n* = 3–4 mice/genotype). Data are presented as mean values ± s.e.m. Data are normalized to *Dag1*^*Control*^ signal in each CA layer

Our results showing that deletion of *Dystroglycan* from PyNs leads to a reduction in CCK+ IN innervation is consistent with previous work [39]. However, the temporal onset of this phenotype has not been determined. During embryonic development, CCK+ INs generated in the caudal ganglionic eminence (CGE) begin populating the hippocampus around E14.5 [59, 60] (Fig. 5A). At postnatal ages, CCK+ INs settle into their final positions within the hippocampus and initially extend axons throughout the hippocampal layers before refining their projections to form characteristic basket synapses onto PyN somas (Fig. 5B, D) [61–63]. We first examined the development of CB_1_R+ terminals in *Dag1*^*Control*^ mice during the first two postnatal weeks (P3-P15), as CB_1_R staining is largely absent from CCK+ INs before birth [64–67]. At early postnatal ages (P3-P5), the majority of CB_1_R+ terminals were observed in the *stratum radiatum* (SR) layer of the hippocampus, where immature PyN dendrites are located (Fig. 5B, D). Between P5 and P10, CB_1_R+ terminals increased in the *stratum pyramidale* (SP) where PyN cell bodies are located. From P15 through adulthood (15 months), CB_1_R+ terminals became progressively concentrated in the SP.

Next, we examined CB_1_R+ terminal development in *Dag1*^*cKO*^ mice. At P3, the earliest age we were able to conclusively identify CCK+ INs, CB_1_R+ staining was already reduced in the hippocampus of *Dag1*^*cKO*^ mice. This reduction persisted throughout the period of postnatal development and into adulthood, as late as 15 months (Fig. 5C, E, F). To further confirm this finding, we stained the hippocampus for VGLUT3, an independent synaptic marker for CCK+ IN terminals that is upregulated during early postnatal ages (Fig. S3A). In *Dag1*^*Control*^ mice, VGLUT3+ terminals increased in the hippocampus during the first two postnatal weeks, and showed a similar pattern of innervation as CB_1_R+ staining (Fig. S3B). In contrast, VGLUT3+ terminals were reduced at all ages examined in *Dag1*^*cKO*^ mice (Fig. S3B). PV staining, which increases between P10 and P15 [68], was unaltered in *Dag1*^*cKO*^ mice compared with controls (Fig. S3C).

**Fig. 6:**
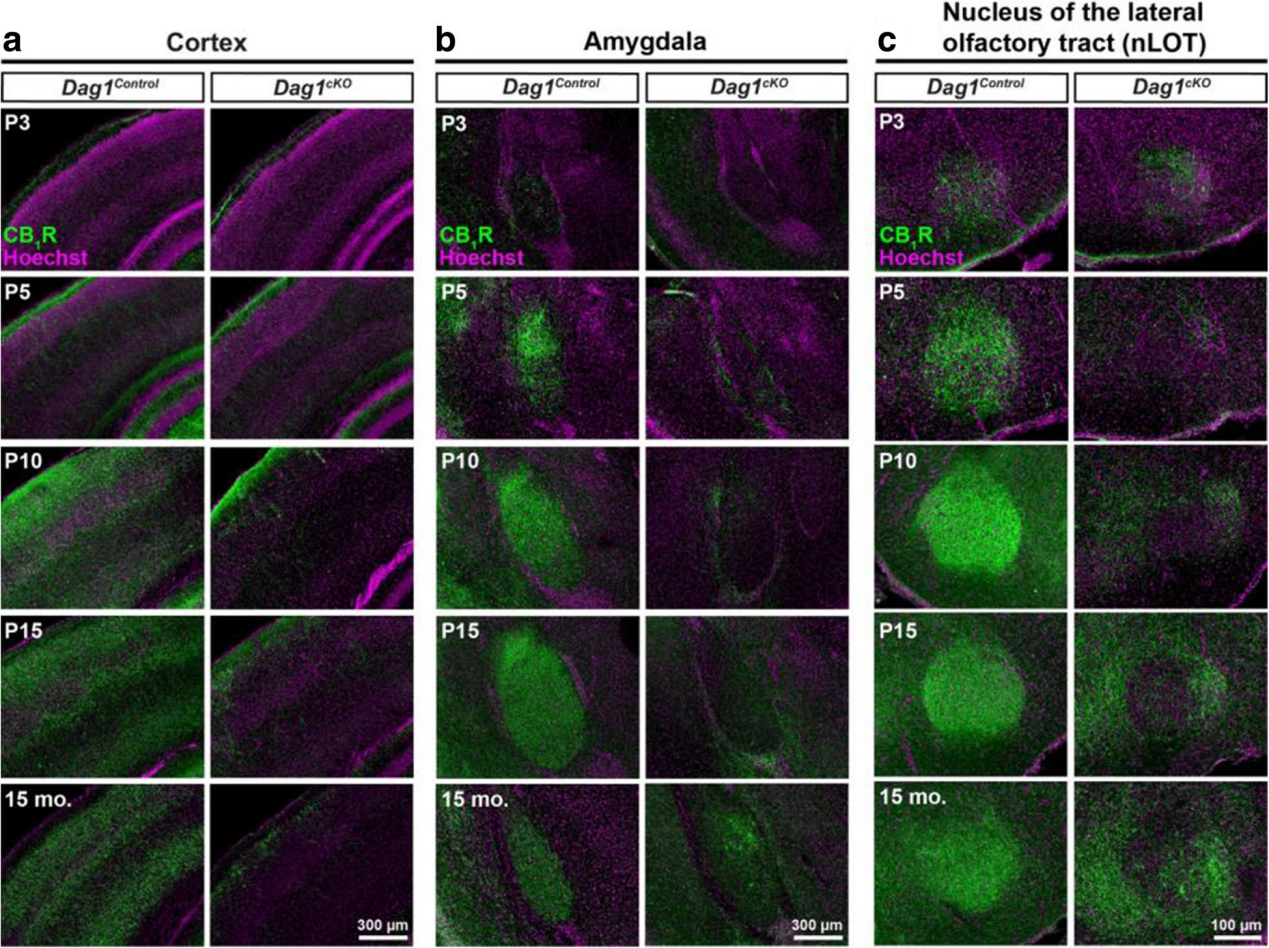
Postnatal development of CCK+ interneurons is impaired in the forebrain of *Dag1*^*cKO*^ mice. (**a-c**) Immunostaining for CB_1_R (green) and Hoechst (magenta) shows the progressive innervation of the cortex (**a**), amygdala (**b**), and nucleus of the lateral olfactory tract (**c**) of *Dag1*^*Control*^ (left panels) mice by CCK+ interneurons from P3-P15. CB_1_R staining is decreased in all regions of *Dag1*^*cKO*^ mice (right panels) at all ages examined from P3–15 months

We next determined whether the reduction of CCK+ INs in the cortex, amygdala, and nLOT followed the same developmental time course as the hippocampus. In *Dag1*^*Control*^ mice, CB_1_R+ terminals gradually increased in density in all regions between P3 and P15, and remained stable beyond this age into adulthood (15 months) (Fig. 6). In contrast, CB_1_R+ terminals in *Dag1*^*cKO*^ mice failed to elaborate during the first two postnatal weeks, and remained sparse in adult animals. Collectively, these results demonstrate that Dystroglycan in PyNs is critical during the first two postnatal weeks for the development and integration of CCK+ INs throughout the forebrain.

### Post-developmental maintenance of CCK+ interneurons does not require Dystroglycan

**Fig. 7:**
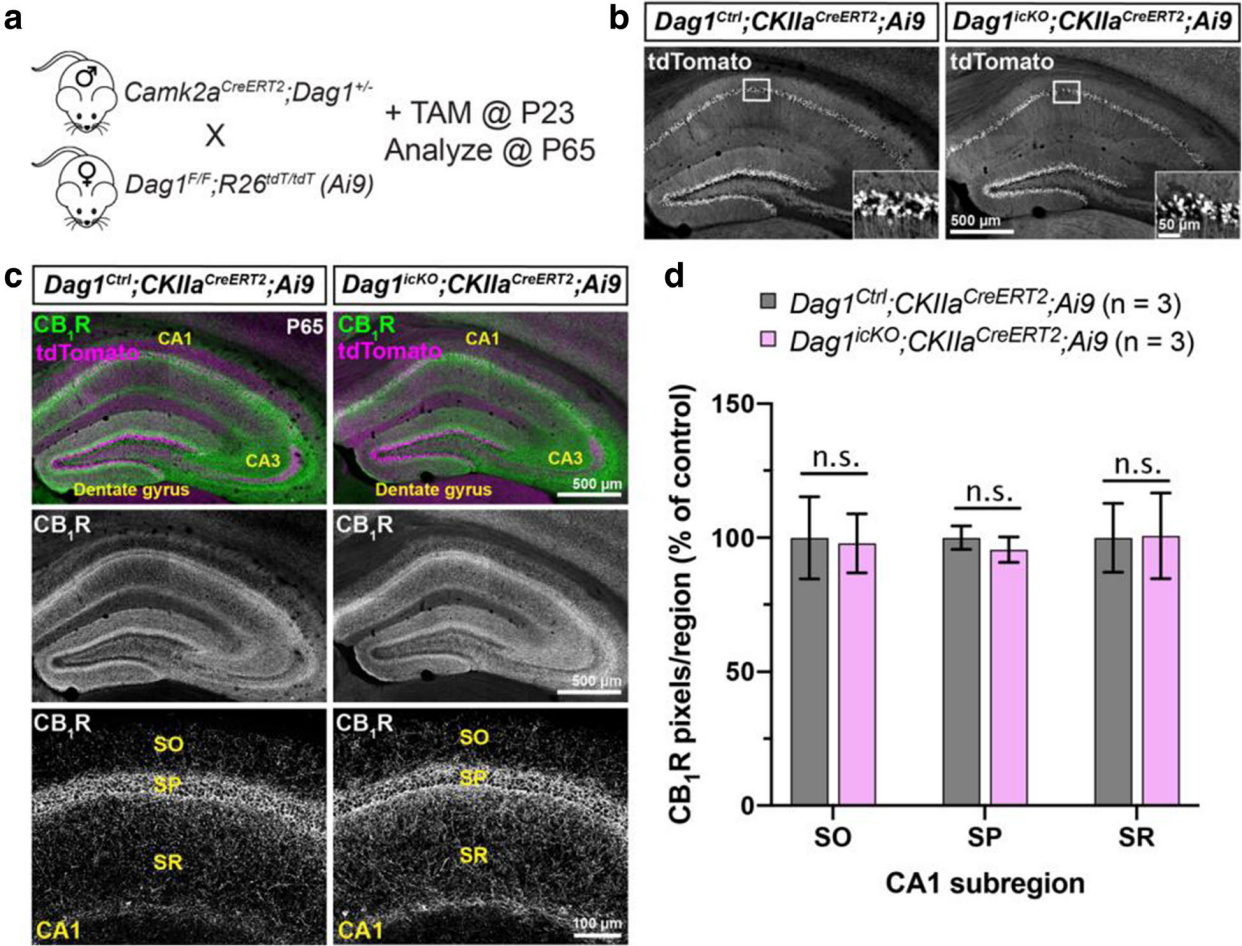
Post-developmental maintenance of CCK+ interneurons does not require Dystroglycan. (**a**) Breeding scheme and experimental approach for generating tamoxifen-inducible *Dystroglycan* conditional knockout mice. *Dag1*^*Ctrl*^*;Camk2a*^*CreERT2*^*;Ai9* and *Dag1*^*icKO*^*;Camk2a*^*CreERT2*^*;Ai9* mice were treated with tamoxifen (5 mg/ml) at P23 and brains were collected for immunohistochemistry 6 weeks later at P65. (**b**) Single channel images of tdTomato staining in the hippocampus show the recombination pattern in PyNs. Insets show enlarged view of tdT+ pyramidal neurons in the CA1. (**c**) Immunostaining for CB_1_R+ terminals (green) and tdTomato signal (magenta) in the hippocampus of P65 *Dag1*^*Ctrl*^*;Camk2a*^*CreERT2*^*;Ai9* (left panels) and *Dag1*^*icKO*^*;Camk2a*^*CreERT2*^*;Ai9* mice (right panels) shows that the deletion of *Dystroglycan* in adult PyNs does not affect CB_1_R+ terminal maintenance. (**d**) Quantification of CB_1_R pixels in hippocampal CA1 of *Dag1*^*Ctrl*^*;Camk2a*^*CreERT2*^*;Ai9* (gray) and *Dag1*^*icKO*^*;Camk2a*^*CreERT2*^*;Ai9* (pink) mice (n.s. = not significant, unpaired two-tailed Student’s t-test; *n* = 3 mice/genotype). Data are presented as mean values ± s.e.m. Data are normalized to *Dag1*^*Control*^ signal in each layer. SO, *stratum oriens*; SP, *stratum pyramidale*; SR, *stratum radiatum*

Inhibitory synaptogenesis increases between P5-P15, and is largely complete by P30 [20, 22, 54]. Therefore, we wanted to assess whether deletion of *Dystroglycan* after inhibitory synapse formation impairs the maintenance of CCK+ INs. To achieve temporal control of *Dystroglycan* deletion from PyNs, we generated mice expressing tamoxifen-inducible *Cre* recombinase under the control of an excitatory neuron-specific promoter *Camk2a*, (Calcium/calmodulin-dependent protein kinase II alpha [69]). Control (*Camk2a*^*CreERT2*^*;DG*^*F/+*^*;Ai9*) or inducible-cKO (*Camk2a*^*CreERT2*^*;DG*^*F/−*^*;Ai9*) mice were administered tamoxifen at P23 via oral gavage, which induced recombination in the majority of PyNs in the hippocampus (Fig. 7A, B). We then analyzed CB_1_R+ innervation 6 weeks later at P65. No differences were found between the *Dag1* inducible-cKO and controls, suggesting that *Dystroglycan* is not required for the post-developmental maintenance of CCK+ INs (Fig. 7C, D).

### Blocking *Bax*-dependent cell death does not rescue CCK+ interneurons in *Dag1*^*cKO*^ mice

**Fig. 8:**
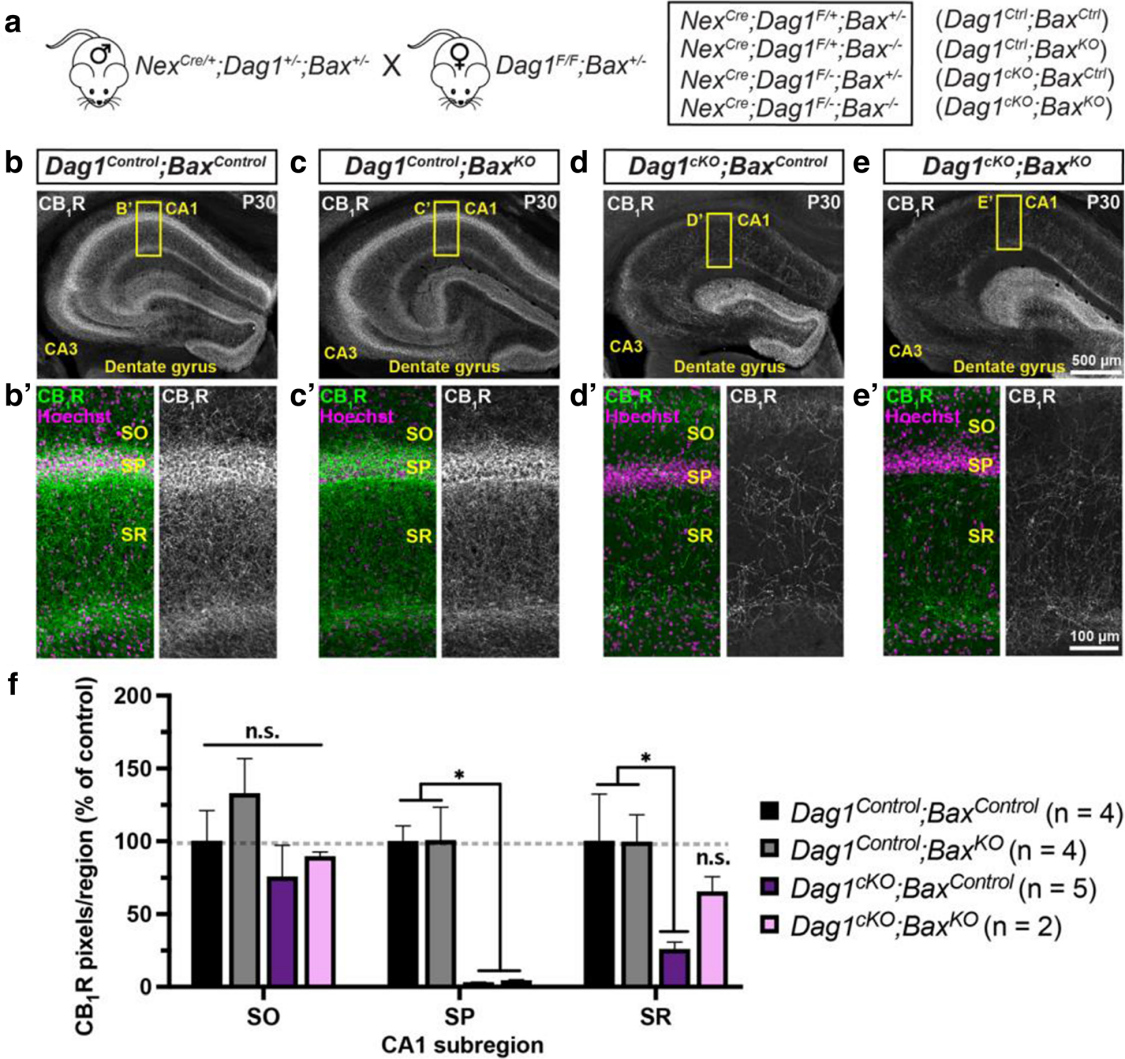
Constitutive deletion of *Bax* does not rescue CB_1_R+ terminals in the hippocampus. (**a**) Breeding scheme for deletion of *Bax* in *Dag1*^*Control*^ and *Dag1*^*cKO*^ mice; the four genotypes analyzed and their abbreviations are shown to the right. (**b-e**) Coronal sections of the hippocampus stained for CB_1_R (gray) from (**b**) *Dag1*^*Control*^*;Bax*^*Control*^, (**c**) *Dag1*^*Control*^;*Bax*^*KO*^, (**d**) *Dag1*^*cKO*^;*Bax*^*Control*^ and (**e**) *Dag1*^*cKO*^;*Bax*^*KO*^ mice. (**B′-E’**) Enlarged images of the CA1 (yellow boxed regions) stained for CB_1_R (green; Right, gray single channel images) and Hoechst (magenta). (**f**) Quantification of CB_1_R pixels in hippocampal CA1 layers from *Dag1*^*Control*^*;Bax*^*Control*^ (black bars), *Dag1*^*Control*^;*Bax*^*KO*^ (gray bars), *Dag1*^*cKO*^;*Bax*^*Control*^ (purple bars), and *Dag1*^*cKO*^;*Bax*^*KO*^ (pink bars) shows that deleting *Bax* fails to rescue the loss of CB_1_R in *Dag1*^*cKO*^ mice (n.s. = not significant; *P < 0.05, unpaired two-tailed Student’s t-test; *n* = 2–5 mice/genotype). Data are presented as mean values ± s.e.m. Data are normalized to *Dag1*^*Control*^*;Bax*^*Control*^ signal in each CA1 layer. SO, *stratum oriens*; SP, *stratum pyramidale*; SR, *stratum radiatum*

The number of PyNs and INs in the forebrain is tightly regulated during early postnatal development, with excess or inappropriately connected neurons pruned by *Bax*-dependent apoptosis [70–73]. PyN apoptosis is largely complete by P5, followed by IN apoptosis which peaks at P7-P9. We hypothesized that in the absence of PyN Dystroglycan, CCK+ INs are unable to recognize their postsynaptic targets and are therefore eliminated by apoptosis. We tested this hypothesis by generating *Dag1*^*Ctrl*^ and *Dag1*^*cKO*^ mice that lack either one (*Dag1*^*Ctrl*^*;Bax*^*Ctrl*^ and *Dag1*^*cKO*^*;Bax*^*Ctrl*^) or both copies of *Bax* (*Dag1*^*Ctrl*^*;Bax*^*KO*^ and *Dag1*^*cKO*^*;Bax*^*KO*^) to block apoptosis (Fig. 8A). Deletion of *Bax* from control mice (*Dag1*^*Ctrl*^*;Bax*^*KO*^) did not alter CB_1_R+ innervation in the CA1 subregion of the hippocampus (Fig. 8B-C, F**)**. In line with our previous results, *Dag1*^*cKO*^*;Bax*^*Ctrl*^ mice lacking one copy of *Bax* had a similar reduction in CB_1_R+ terminals as *Dag1*^*cKO*^ mice (Fig. 8D, F). Surprisingly, we found that complete deletion of *Bax* in *Dag1*^*cKO*^ mice (*Dag1*^*cKO*^*;Bax*^*KO*^) was not sufficient to rescue CB_1_R+ innervation (Fig. 8E, F). Staining for an additional CCK+ IN synapse marker (VGLUT3) further confirmed this result (Fig. S4). Finally, we examined whether deletion of *Bax* could rescue CB_1_R+ innervation in the cortex, amygdala, and the nucleus of the lateral olfactory tract (nLOT) of *Dag1*^*cKO*^ mice (Fig. S5). In all regions examined, CB_1_R+ terminals were reduced in mice lacking *Dystroglycan* (*Dag1*^*cKO*^*;Bax*^*Ctrl*^). Similar to our observations in the hippocampus, deleting both copies of *Bax* (*Dag1*^*cKO*^*;Bax*^*KO*^) was not sufficient to rescue CB_1_R+ innervation in the cortex, amygdala, or nLOT (Fig. S5). Collectively, these results suggest that loss of CB_1_R+ innervation in the absence of PyN Dystroglycan is not due to CCK+ INs undergoing *Bax*-dependent apoptosis.

### CCK+ interneurons inappropriately innervate the striatum of *Dag1*^*cKO*^ mice

**Fig. 9:**
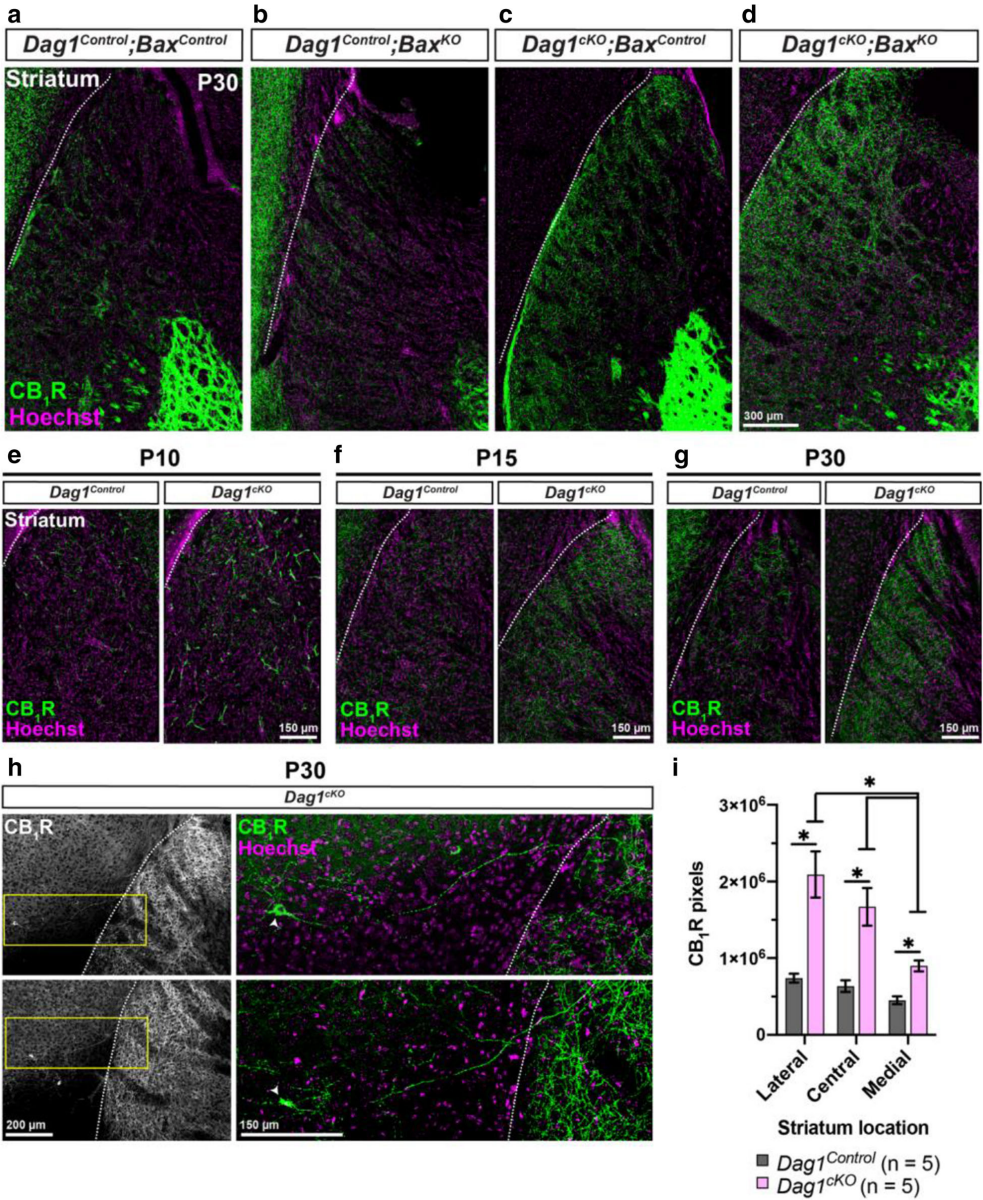
CCK+ interneurons inappropriately innervate the striatum of *Dag1*^*cKO*^ mice. (**a-d**) Immunostaining for CB_1_R (green) and Hoechst (magenta) shows minimal CB_1_R innervation in the striatum of P30 (**a**) *Dag1*^*Control*^*;Bax*^*Control*^ and (**B**) *Dag1*^*Control*^;*Bax*^*KO*^ mice. Striatal innervation by CB_1_R+ axons is abnormally increased in (**c**) *Dag1*^*cKO*^;*Bax*^*Control*^ and (**d**) *Dag1*^*cKO*^;*Bax*^*KO*^ mice. (**e-g**) Immunostaining for CB_1_R (green) and Hoechst (magenta) in the striatum of *Dag1*^*Control*^ and *Dag1*^*cKO*^ mice at P10 (**e**), P15 (**f**), and P30 (**g**), showing that the inappropriate CB_1_R innervation in the striatum of *Dag1*^*cKO*^ mice increases gradually between P10-P30. (**h**) Low magnification images (10X) of CB_1_R+ cell bodies and their axons (Left panels, gray) near the cortico-striatal boundary from two separate *Dag1*^*cKO*^ mice at P30. Yellow boxed regions (right panels) show high magnification (20X) images of individual CB_1_R+ cell bodies (arrowheads, green) and their axons projecting from the cortex into the striatum. White dotted lines (**a-h**) indicate the approximate boundary between the cortex and striatum. (**i**) Quantification of CB_1_R pixels in the caudal striatum from P30 *Dag1*^*Control*^ (black bars) and *Dag1*^*cKO*^ (pink bars) mice shows increased CB_1_R staining in *Dag1*^*cKO*^ (*P < 0.05, unpaired two-tailed Student’s t-test; *n* = 5 mice/genotype). Data are presented as mean values ± s.e.m.

During embryonic development, CCK+ INs are produced in and migrate through the caudal ganglionic eminence (CGE), one of two ventral forebrain regions that ultimately develop into the striatum. Expression of *Dystroglycan* in striatal neurons is retained in *Dag1*^*cKO*^ mice, as they are not targeted by *Nex*^*Cre*^ (Fig. 4A-B [39, 41]). We therefore examined CB_1_R innervation of the striatum in *Dag1*^*Control*^ and *Dag1*^*cKO*^ mice. In *Dag1*^*Control*^ mice, CB_1_R innervation in the striatum was present, but sparse compared with neighboring regions of the cortex (Fig. 9A) [74, 75]. In contrast, CB_1_R innervation in the striatum of *Dag1*^*cKO*^ mice was noticeably increased (Fig. 9C, I). The lateral regions of the striatum closest to the cortex exhibited dense CB_1_R innervation, which decreased towards the medial striatum. Global deletion of *Bax* from *Dag1*^*Control*^ or *Dag1*^*cKO*^ mice did not alter the pattern of CB_1_R innervation in the striatum (Fig. 9B, D).

Examination of the developmental timecourse of CB_1_R+ innervation in the striatum revealed sparse CB_1_R+ terminals at P10 in both *Dag1*^*Control*^ and *Dag1*^*cKO*^ mice (Fig. 9E), which increased in *Dag1*^*cKO*^ mice compared with controls between P15 and P30 (Fig. 9F-G). This coincides with the period of CB_1_R+ innervation of forebrain targets in *Dag1*^*Control*^ mice. Occasionally, CB_1_R+ cell bodies could be seen in the cortex near the striatal boundary, with their axon terminals projecting into the striatum (Fig. 9H). These results suggest that some CCK+ INs in the cortex of *Dag1*^*cKO*^ mice may redirect their axons into the neighboring regions of the striatum that retain Dystroglycan.

## Discussion

Dystroglycan plays a critical role in maintaining the integrity of the neuroepithelial scaffold during early stages of brain development, which has made it difficult to assess its function within neurons at subsequent stages. In the current study, we show that Dystroglycan in pyramidal neurons regulates the development of a subset of their pre-synaptic partners. When *Dystroglycan* is selectively deleted from PyNs, CCK+ INs throughout the entire forebrain fail to properly integrate, and largely disappear during the first postnatal week. Surprisingly, we found that deletion of *Bax* did not rescue CCK+ INs in *Dag1*^*cKO*^ mice, suggesting their disappearance is not due to apoptotic cell death. The few remaining CCK+ INs redirect their axons into neighboring regions of the brain in which Dystroglycan is still present, suggesting that Dystroglycan functions as a part of a synaptic partner recognition complex.

### What stage of CCK+ interneuron development requires Dystroglycan?

The localization of Dystroglycan to inhibitory synapses in forebrain pyramidal neurons has been described by multiple studies, while its function at these synapses has remained obscure [36–38, 76]. Recently, it was found that Dystroglycan is required for the formation and function of CCK+ inhibitory basket synapses, but not PV+ basket synapses onto the same PyNs [39]. This finding is significant, because very little is known about the molecules and mechanisms involved in orchestrating the formation of specific subtypes of inhibitory synapses [17]. However, since the earliest timepoint examined in this previous study was P21, it was unclear what stage of synapse development requires Dystroglycan.

During neural circuit development, neurons must first migrate and direct their axons to their appropriate targets, then recognize the appropriate synaptic partners from a myriad of potential choices, then finally form functional synapses [13]. Our data suggest that Dystroglycan is required for the least understood of these processes: synaptic partner recognition. This is supported by the observation that CCK+ INs are present at the earliest stages they can be conclusively identified in the forebrain of *Dag1*^*cKO*^ mice (P3), but then fail to elaborate their axons and integrate into these circuits during the first postnatal week (Figs. 5, S3, Fig. 6). Interestingly, the few remaining CCK+ INs appear to project their axons into regions that continue to express *Dystroglycan* in *Dag1*^*cKO*^ mice **(**Figs. 5, 9**)**. Taken together, this data suggests that CCK+ INs in *Dag1*^*cKO*^ mice fail to recognize their normal postsynaptic PyN targets in the hippocampus and cortex during early postnatal development, and instead re-route to neighboring *Dag1*+ neurons, discussed further below.

The process of synaptic partner recognition in mammals has been difficult to study due to our inability to precisely identify and genetically manipulate the specific neuronal populations during development. Determining whether loss of Dystroglycan impairs CCK+ IN development before birth is technically challenging due to the lack of immunohistochemical and genetic tools for detecting CCK+ INs prenatally [60]. The cannabinoid receptor-1 (*Cnr1*) and cholecystokinin (*Cck*) genes are both expressed in PyNs at prenatal timepoints, limiting their usefulness for detecting CCK+ INs. Transcription factors such as Prox1 are also of limited usefulness due to its broad expression in multiple CGE-derived IN subtypes [77]. VGLUT3, which labels a subset of CCK+ INs, does not increase in expression until the first postnatal week [54]. Other IN subtypes exhibit delayed expression of selective molecular markers as well. For instance, MGE-derived Parvalbumin INs do not begin to express Parvalbumin until P10, well after the period of initial synaptic partner recognition [68, 78].

### What happens to CCK+ interneurons in the absence of Dystroglycan?

Our results show that deletion of *Dystroglycan* from PyNs resulted in a loss of all of the markers we used to identify CCK+ INs in the forebrain (Figs. 2, 3, 4). What happens to these neurons in the absence of PyN Dystroglycan? One possibility that we examined is that CCK+ INs undergo apoptosis. During the first 2 weeks of development (P5-P10), a significant number of excitatory and inhibitory neurons are pruned by *Bax*-dependent apoptotic cell death [70–73]. This ensures the proper number of neurons and removes neurons that fail to integrate into the developing circuit. Whereas *Bax*-dependent developmental cell death has been described for most MGE and CGE-derived interneuron subtypes, whether CCK+ INs normally undergo the same process has not been directly examined [72, 73]. We tested whether the loss of CCK+ INs in *Dag1*^*cKO*^ mice could reflect premature or amplified developmental apoptosis, which peaks around P9 for other IN subtypes. However, constitutive deletion of *Bax*, which is sufficient to block developmental apoptosis in other neuronal populations, did not rescue CCK+ INs (Figs. 8, S4, S5). This suggests that canonical apoptosis is not responsible for the loss of CCK+ INs in *Dag1*^*cKO*^ mice. It is possible that CCK+ INs are eliminated in a *Bax*-independent manner, similar to some populations of Cajal-Retzius cells in the cortex and astrocytes in the developing retina [79, 80].

CCK+ INs comprise a molecularly and morphologically diverse group of cells that include both cell body targeting (perisomatic) and multiple dendrite targeting subtypes [54, 81, 82]. In the hippocampus, CCK+ INs frequently express one of two non-overlapping markers, VGLUT3 (~ 45%) and VIP (~ 16%) [53]. In *Dag1*^*cKO*^ mice, all synaptic and cell body markers selective for CCK+ INs (CB_1_R, VGLUT3, NECAB1) that we examined were reduced at the onset of their expression. While it is formally possible that Dystroglycan in PyNs is required for CCK+ INs to fully differentiate into their mature, molecularly defined subtype, we consider this unlikely. In this situation, Dystroglycan present on PyNs would be required to transmit a retrograde signal to CCK+ INs to direct their differentiation. We are unaware of any cell adhesion molecules that function in this manner. Rather, fate switching or failure to fully differentiate is usually observed upon cell-autonomous loss of specific transcription factors [83].

Our data also indicate that Dystroglycan is not required to maintain CCK+ INs after the period of synapse formation (Fig. 7). This is in contrast to a previous study that showed a gradual reduction in the number of Vglut3+ puncta when *Dag1* was deleted in adult mice using AAV-Cre [39]. Aside from the different approaches used for adult deletion, this difference may arise from the level of analysis: in our study, we saw no difference in the cellular organization of CCK+ INs following adult deletion, whereas the previous study was focused specifically on presynaptic puncta. It is possible that in our inducible-cKO (*Camk2a*^*CreERT2*^*;DG*^*F/−*^*;Ai9*) mice, synaptic inputs from CCK+ INs are reduced without altering the survival of these neurons. Alternatively, there may still be some residual Dystroglycan protein remaining in *Camk2a*^*CreERT2*^*;DG*^*F/−*^*;Ai9* mice, at levels sufficient to support CCK+ IN maintenance.

Although CCK+ INs and their terminals were dramatically reduced throughout the brains of *Dag1*^*cKO*^ mice, some CCK+ IN terminals were still present, particularly along the cortico-striatal boundary and in the upper dendritic layers of the cortex (layer 1) and hippocampus. Importantly, striatal neurons and Cajal-Retzius cells, which are located in superficial cortical layers during postnatal development, are not targeted by *Nex*^*Cre *^[84]. This suggests that in the absence of *Dystroglycan* on their normal postsynaptic targets (PyNs), CCK+ INs may direct their axons to secondary synaptic targets that retain *Dystroglycan* expression.

A number of studies have indicated that synaptic partner recognition and targeting may be “stringent” or “flexible”, depending on the cell type involved. Studies in the *Drosophila* visual system have shown that synaptic cell adhesion molecules such as DIP/Dprs can promote either stringent or flexible outcomes among synaptic partners depending on the cellular context and the molecules involved. For instance, postsynaptic Dm8 neurons containing the receptor DIP-γ undergo cell death if not innervated by a matching R7 photoreceptor containing the cognate ligand Dpr11 [85]. In contrast, loss of DIP-β from L4 neurons does not impair synapse formation or cause cell death, but instead leads to ectopic synapses onto alternative synaptic partners [86]. Synaptic partner recognition “flexibility” and “stringency” has also been demonstrated in the mammalian nervous system. In the developing retina, On-alpha retinal ganglion cells will re-wire to increase inputs from neighboring bipolar cell types when their normal presynaptic inputs (Type 6 bipolar cells) are genetically ablated [87]. In contrast preGABA INs in the developing spinal cord retract their processes when their primary targets (proprioceptor axons) are not present, rather than forming synapses onto secondary targets [88]. Despite retracting their axons, preGABA INs do not undergo cell death, suggesting that loss of neurons is not a necessary consequence of losing synaptic partners. In *Dag1*^*cKO*^ mice, CCK+ INs may stringently require *Dystroglycan* for their ability to recognize their primary synaptic targets and die in a *Bax*-independent manner in its absence. The observation that some CCK+ INs near the cortico-striatal boundary survive and innervate the striatum suggests that they may exhibit some degree of flexibility to make contacts onto secondary targets. Determining whether the remaining CCK+ INs in *Dag1*^*cKO*^ mice exhibit normal morphological and physiological properties will require fate mapping these neurons, which is difficult with currently available genetic tools.

### Why are CCK+ interneurons selectively affected in *Dag1*^*cKO*^ mice?

CCK+ INs appear to be the only interneuron subtype affected by deletion of *Dystroglycan* from PyNs. Compared to other IN populations, CCK+ INs express high levels of CB_1_Rs, which can play important roles in neuronal proliferation, migration, and axon outgrowth [89]. In utero exposure to exogenous cannabinoids results in a specific loss of CCK+ INs through unknown mechanisms [90]. However, conditional deletion of the cannabinoid receptor-1 gene *Cnr1* from CCK+ INs does not affect interneuron migration or neurochemical specification, but rather increases the number of perisomatic VGLUT3+ inhibitory synapses on cortical PyNs [67]. In addition, CB_1_R signaling is not necessary for the survival of CCK+ INs [91]. Therefore, it is unlikely that alterations in CB_1_R activity underlie the selective loss of CCK+ INs in *Dag1*^*cKO*^ mice.

One possible explanation for this selective loss is that Dystroglycan interacts with specific molecules on presynaptic CCK+ INs compared with other IN subtypes. Dystroglycan is highly glycosylated, and unique matriglycan moieties present on its extracellular domain bind to proteins containing Laminin G (LG) domains [92]. Proteins that bind Dystroglycan through their LG domains include extracellular matrix proteins (Agrin, Laminins, Perlecan), axon guidance molecules (Slits, Celsr3), as well as synaptic proteins (Neurexin, Pikachurin) [29, 33, 93–98]. Several other putative synaptic proteins also contain LG domains (ie: Cntnap1–6), although their binding to Dystroglycan has not been examined.

Biochemical experiments have identified α-DG as a major interaction partner of α- and β-neurexins in whole brain lysates, and these interactions are dependent upon the lack of splice inserts in LNS2 and LNS6 of neurexin [98–101]. Conditional deletion of all three *Neurexins* from interneurons revealed distinct outcomes depending on the IN population examined [102]. Deletion of all *Neurexin* isoforms from PV+ INs results in a significant decrease in the number of PV+ synapses in the cortex, whereas it does not affect inhibitory synapse numbers when deleted from SST+ INs. While PV+ IN numbers were not affected by conditional deletion of *Neurexins*, this could reflect the timing of deletion, which is unlikely to occur before three weeks of age based on the onset of *Cre* expression [68, 78]. Nrxn1α and Neurexin 3α/β are expressed at significantly higher levels in CCK+ INs than in PV+ INs, and CCK+ INs predominantly express Neurexin isoforms lacking splice inserts in LNS6 [100]. Therefore, CCK+ INs may show a larger degree of Nrxn:Dystroglycan interaction than other IN subtypes. Mice harboring a mutation in *Dystroglycan* that exhibits reduced glycosylation, and thus Neurexin binding capacity (*Dag1*^*T190M*^), showed no impairments in CCK+ IN terminal development [39, 103]. However, these mice do not display the cortical migration phenotypes associated with a complete loss of *Dystroglycan*, suggesting that Dystroglycan retains some residual function, which may be sufficient for CCK+ IN terminal development. Whether Neurexins are required cell autonomously in CCK+ INs for their development has not been directly tested, in part due to a lack of genetic tools.

### Limitations in studying CCK+ interneuron development

Our understanding of CCK+ IN development and function has lagged behind other interneuron subtypes (PV, SOM, VIP, etc) due in part to the lack of viral and mouse genetic tools available for selectively labeling and manipulating CCK+ INs. All major markers of CCK+ INs (CCK, CB1R, and VGLUT3) are also expressed at lower levels in PyNs, limiting the usefulness of single promoter/recombinase approaches for targeting CCK+ INs [104–106]. Specific targeting of CCK+ INs therefore requires dual recombinase-based intersectional approaches, including CCK-Cre;Dlx5/6-Flp double transgenic mice [107–111], dual CCK-dsRed;GAD67-GFP reporter mice [60], or *VGLUT3*^*Cre*^ mice which label approximately half of CCK+ INs [54, 112]. Other reporter lines (*5HT3AR*^*EGFP*^) target the entire CGE-derived interneuron population, of which CCK+ INs only comprise ~ 10% [113, 114]. A recently developed *Sncg*^*FlpO*^ mouse line appears to provide selective genetic access CCK+ basket cells by taking advantage of the fact that *Sncg* is specifically expressed in CCK+ INs [115]. However, it is not clear when the onset of recombination occurs in this line, and whether it will be useful for studying the early development of CCK+ INs. Indeed, many of the genes used for targeting IN subtypes are not significantly expressed until after the first postnatal week in mice, when much of the process of synaptic partner recognition and initial synapse formation has already occurred (Fig. S3 [54, 68, 78]).

## Conclusion

In this study, we identified a critical role for excitatory neuron Dystroglycan in regulating the development of forebrain CCK+ interneurons during the first postnatal week. Given the emerging role for CCK+ INs and cannabinoid signaling in controlling neural circuit activity, *Dag1*^*cKO*^ mice may be useful for studying the consequences of losing a major IN population.

## Materials and methods

### Animal husbandry

All animals were housed and cared for by the Department of Comparative Medicine (DCM) at Oregon Health and Science University (OHSU), an AAALAC-accredited institution. Animal procedures were approved by OHSU Institutional Animal Care and Use Committee (Protocol # IS00000539) and adhered to the NIH *Guide for the care and use of laboratory animals*. Animals older than postnatal day 6 (P6) were euthanized by administration of CO_2,_ animals <P6 were euthanized by rapid decapitation. Animal facilities are regulated for temperature and humidity and maintained on a 12 h light-dark cycle and animals were provided food and water ad libitum.

### Mouse strains and genotyping

The day of birth was designated postnatal day 0 (P0). Ages of mice used for each analysis are indicated in the figure and figure legends. Mice were maintained on a C57BL/6 background and have been previously described or obtained from JAX (Table 1): *Dystroglycan* conditional mice (*Dag1*^*Flox*^) [30, 117], *Nex*^*Cre*^ [41, 42], *VGLUT3*^*Cre*^ [118], *Bax*^*−/−*^ [119, 120], *Camk2a*^*CreERT2*^ [69], *Ai9*^*LSL-tdTomato*^ [69], and *R26*^*LSL-H2B-mCherry*^ [44]. Loss of BAX protein in *Bax*^*−/−*^ mouse brains was validated by western blot in a previous study [121]. Genomic DNA extracted from tissue samples (Quanta BioSciences) was used to genotype animals. The presence of the Cre allele in *Nex*^*Cre*^ mice and *Camk2a*^*CreERT2*^ mice was detected using generic Cre primers (JAX).

**Table 1:** Mouse strains

Common name	Strain name	Reference	Stock #
*Dag1*^*−/−*^	*B6.129-Dag1*^*tm1Kcam*^*/J*	[116]	006836
*Dag1*^*Flox*^	*B6.129(Cg)-Dag1*^*tm2.1Kcam*^*/J*	[117]	009652
*Nex*^*Cre*^	*NeuroD6*^*tm1(cre)Kan*^	[41]	MGI:4429523
*Vglut3*^*Cre*^	*Tg(Slc17a8-icre)1Edw*	[118]	018147
*Ai9*^*LSL-tdTomato*^	*B6.Cg-Gt(ROSA)26Sor*^*tm9(CAG-tdTomato)Hze*^*/J*	[69]	007909
*Camk2a*^*CreERT2*^	*B6.Tg(Camk2a-cre/ERT2)1Aibs/J*	[69]	012362
*R26*^*LSL-H2B-mCherry*^	*B6.Gt(ROSA)26Sor*^*tm1.1Ksvo*^	[44]	023139
*Bax*^*−/−*^	*B6.129X1-Bax*^*tm1Sjk*^*/J*	[119]	002994

### Tamoxifen administration

Tamoxifen (Sigma; Cat# T5648-1G) was dissolved 1:10 in sunflower seed oil. Each mouse was orally gavaged with 200 μL of tamoxifen at a final concentration of 5 mg/ml tamoxifen.

### Perfusions and tissue preparation

Brains from mice younger than P15 were dissected and fixed in 4% paraformaldehyde (PFA) in phosphate buffered saline (PBS) overnight for 18–24 h at 4 °C. Mice P15 and older were deeply anesthetized using CO2 and transcardially perfused with ice cold 0.1 M PBS for two minutes to clear blood from the brain, followed by 15 mL of ice cold 4% PFA in PBS. After perfusion, brains were dissected and post-fixed in 4% PFA for two hours. Brains were rinsed with PBS, embedded in 4% low-melt agarose (Fisher: Cat# 16520100), and 50 μm sections were cut on a vibratome (VT1200S, Leica Microsystems Inc., Buffalo Grove, IL).

### Immunohistochemistry and antibodies

Single and multiple immunofluorescence detection of antigens was performed as follows: Free-floating vibratome sections (50 μm) were briefly rinsed with PBS, then blocked for 1 h in PBS containing 0.2% Triton-X (PBST) plus 10% normal donkey serum. Sections were incubated with primary antibodies (Table 2) diluted in PBST at 4 °C overnight (18–24 h) or for 3 days for Dystroglycan staining. The following day, sections were rinsed briefly with PBS, then washed with PBST three times for 20 min each. Sections were then incubated with a cocktail of secondary antibodies (1:1000, Alexa Fluor 488, 546, 647; Fisher) in PBST for 90 min at room temperature. Sections were washed with PBS three times for 20 min each and counterstained with Hoechst 33342 (Life Technologies, Cat# H3570) for 10 min to visualize nuclei. Finally, sections were mounted on slides using Fluoromount-G (Fisher; SouthernBiotech) and sealed using nail polish.

**Table 2:** Primary antibodies used for immunohistochemistry

Target	Host species	Dilution	Source	Catalog #	RRID
α-Dystroglycan (IIH6C4)	Mouse	1:200	Millipore	05–593	AB_309828
Calbindin	Rabbit	1:4000	Swant	CB38	AB_10000340
Calretinin	Rabbit	1:4000	Swant	CG1	AB_2619710
CB1R	Guinea pig	1:2000	Synaptic Systems	258–104	AB_2661870
Cux1	Rabbit	1:250	Santa Cruz Biotech	sc-13,024	AB_2261231
GFAP	Mouse	1:1000	Millipore	MAB360	AB_2109815
NECAB1	Rabbit	1:2000	Sigma	HPA023629	AB_1848014
Parvalbumin	Goat	1:2000	Swant	PVG-213	AB_2650496
Somatostatin	Rabbit	1:2000	Peninsula Labs	T-4103	AB_518614
tdTomato	Goat	1:1000	Biorbyt	orb182397	AB_2687917
VGlut3	Rabbit	1:2000	Synaptic Systems	135–203	AB_887886

### Microscopy

Imaging was performed on a Zeiss Axio Imager M2 fluorescence upright microscope equipped with an Apotome.2 module for structured illumination microscopy. The microscope uses a metal halide light source (HXP 200 C), Axiocam 506 mono camera, and 10X/0.3 NA EC Plan-Neofluar, 20X/0.8 NA Plan-Apochromat objectives. Z-stack images were acquired and processed as maximum projection images using Zeiss Zen Imaging software, and analyzed offline in ImageJ/FIJI [122]. Images used for quantification between genotypes were acquired using the same exposure times. Brightness and contrast were adjusted in FIJI to improve visibility of images for publication. Figures were composed in Adobe Illustrator CS6 (Adobe Systems).

### Quantification

Quantification of CB_1_R terminals in the hippocampus (CA1, CA3, Dentate gyrus) and caudal striatum was performed on 5 μm z-stacks acquired using a 20X objective. Six to twelve sections per animal (technical replicates) from at least three animals per genotype (biological replicates) were used for analysis, except where noted in the text and figure legends. Sections were taken from equivalent rostro-caudal positions including the dorsal hippocampus (Bregma between − 1.48 to − 1.94 mm) using coordinates from the mouse brain atlas (Franklin and Paxinos, 1997). All images used for quantification were processed identically. Briefly, background subtraction (Rolling ball radius = 50) and mean filtering (Smooth function in FIJI) were applied to each image to enhance the detection of CB_1_R terminals by thresholding. To measure CB_1_R signal in specific regions of interest (ROIs), a threshold was manually set and applied equally across images to detect only CB_1_R signal. Separate regions of interest (ROIs) were used to quantify CB_1_R pixels in CA1 and CA3 layers: stratum oriens (SO), stratum pyramidale (SP) and stratum radiatum (SR). Three separate ROIs were used to analyze Dentate gyrus layers: Outer molecular layer (OML), Inner molecular layer (IML), and Granule cell layer (GCL). Hoechst signal in the SP (CA regions) and GCL (Dentate regions) were used to align the ROIs consistently for each image. Raw integrated density values from each ROI were averaged across all images for each animal and normalized to the mean intensity of the control group (set to 100% for each ROI).

### Experimental design and statistical analysis

All phenotypic analyses were conducted using tissue collected from at least three mice per genotype from at least two independent litters unless otherwise noted. The number of mice used for each analysis (“n”) are indicated in the figures and figure legends. No specific power analyses were performed, but sample sizes were similar to our previous work and other published literature [28, 29, 33]. Male and female mice were analyzed together. In many cases, highly penetrant phenotypes revealed the genotypes of the mice and no blinding could be performed. Significance between groups was determined using unpaired two-tailed Student’s t-test. Data are presented as mean ± standard error of the mean (s.e.m) and statistical significance was set at alpha = 0.05 (*P* < 0.05). Graphical representations of data and statistical analyses were performed in GraphPad Prism 8 (San Diego, CA).

## Supplementary Information

**Additional file 1: Fig. S1**. *Nex*^*Cre*^ drives recombination in forebrain pyramidal neurons but not interneurons or glia. (A) Coronal sections from *Nex*^*Cre*^;*R26*^*LSL-H2B-mCherry*^ reporter mice at P21 show mCherry+ nuclei (magenta) of pyramidal neurons in the hippocampus, cortex, amygdala, and nucleus of the lateral olfactory tract (nLOT). (B) Hippocampal sections from *Nex*^*Cre*^;*R26*^*LSL-H2B-mCherry*^ reporter mice immunostained for interneuron markers (green) Calbindin (left panels), Parvalbumin (middle panel), and CB_1_R (right panel) show no overlap of interneuron cell bodies with mCherry+ nuclei. White arrowheads indicate CB_1_R+ cell bodies. SO, *stratum oriens*; SP, *stratum pyramidale*; SR, *stratum radiatum*. (C) The astrocyte marker GFAP (green) shows no overlap with mCherry+ nuclei in the hippocampal CA regions or dentate gyrus (left and middle panels). Inset (middle panel) shows a magnified view of astrocyte nuclei (blue). mCherry+ nuclei occupy the outer third of the dentate gyrus granule cell layer (right panel). ML, molecular layer; GCL, granule cell layer.

**Additional file 2: Fig. S2**. CCK+ interneuron innervation of the dentate gyrus is minimally altered in *Dag1*^*cKO*^ mice. (A) Immunostaining of CB_1_R in the dentate gyrus from P30 *Dag1*^*Control*^ (left panels) and *Dag1*^*cKO*^ mice (right panels). Single channel images of CB_1_R (gray) are shown below. (B) Quantification of CB_1_R pixels for each dentate gyrus layer (*P < 0.05, unpaired two-tailed Student’s t-test; *n* = 4 mice/genotype). Data are presented as mean values ± s.e.m. Data are normalized to *Dag1*^*Control*^ signal in each dentate gyrus layer. OML, outer molecular layer; IML, inner molecular layer; GCL, granule cell layer.

**Additional file 3: Fig. S3**. CCK+ interneuron markers are reduced postnatally in *Dag1*^*cKO*^ mice. (A) Images of hippocampal CA1 from *VGLUT3*^*Cre*^*;Ai9* mice from P3-P18. Immunostaining for tdTomato (green) shows progressive increase in VGLUT3 expression in the pyramidal cell layer (SP, magenta). (B) Immunostaining for VGLUT3 in the CA1 of *Dag1*^*Control*^ (top panels) and *Dag1*^*cKO*^ mice (bottom panels) from P3-P15. Note the lack of VGLUT3 expression at all ages in *Dag1*^*cKO*^ mice. (C) Parvalbumin (PV) labeling is similar in the CA1 of *Dag1*^*Control*^ (top panels) and *Dag1*^*cKO*^ mice (bottom panels) from P5-P30.

**Additional file 4: Fig. S4**. Constitutive deletion of *Bax* in *Dag1*^*cKO*^ mice does not rescue VGLUT3+ terminals. (A-D) Coronal sections of the hippocampus stained for VGLUT3 (gray) from P30 (A) *Dag1*^*Control*^*;Bax*^*Control*^, (B) *Dag1*^*Control*^;*Bax*^*KO*^, (C) *Dag1*^*cKO*^;*Bax*^*Control*^ and (D) *Dag1*^*cKO*^;*Bax*^*KO*^ mice. (A’-D′) Magnified images of the CA1 (yellow boxed regions) stained for VGLUT3 (green; Right, gray single channel images) and Hoechst (magenta) to stain the pyramidal cell layer (SP). SO, *stratum oriens*; SP, *stratum pyramidale*; SR, *stratum radiatum*.

**Additional file 5: Fig. S5**. Constitutive deletion of *Bax* in *Dag1*^*cKO*^ mice does not rescue CB_1_R+ terminals in the forebrain. (A-C) Coronal sections immunostained for CB_1_R (green) and Hoechst (magenta) in the cortex (A), amygdala (B), and nucleus of the lateral olfactory tract (C) of P30 *Dag1*^*Control*^*;Bax*^*Control*^, *Dag1*^*Control*^;*Bax*^*KO*^, *Dag1*^*cKO*^;*Bax*^*Control*^ and *Dag1*^*cKO*^;*Bax*^*KO*^ mice.

## Data Availability

The datasets used during the current study are available from the corresponding author upon request.

## Abbreviations

BAX: BCL2-associated X protein
CAM: Cell adhesion molecule
CB_1_R: Cannabinoid receptor 1
CCK: Cholecystokinin
CGE: Caudal ganglionic eminence
cKO: Conditional knockout
CNS: Central nervous system
GABA: Gamma-aminobutyric acid
IN: Interneuron
MGE: Medial ganglionic eminence
P: Postnatal day
PV: Parvalbumin
PyN: Pyramidal neuron
SST: Somatostatin
VGLUT3: Vesicular glutamate transporter 3
