# Supplementary material for: Neuronal Dystroglycan regulates postnatal development of CCK/cannabinoid receptor-1 interneurons
Source: Neural Dev. 2021 Aug 6;16:4. doi: 10.1186/s13064-021-00153-1 (PMC8349015; doi:10.1186/s13064-021-00153-1)
Supplement: Supplementary file 1 — Additional file 1: Fig. S1. NexCre drives recombination in forebrain pyramidal neurons but not interneurons or glia. (A) Coronal sections from NexCre;R26LSL-H2B-mCherry reporter mice at P21 show mCherry+ nuclei (magenta) of pyramidal neurons in the hippocampus, cortex, amygdala, and nucleus of the lateral olfactory tract (nLOT). (B) Hippocampal sections from NexCre;R26LSL-H2B-mCherry reporter mice immunostained for interneuron markers (green) Calbindin (left panels), Parvalbumin (middle panel), and CB1R (right panel) show no overlap of interneuron cell bodies with mCherry+ nuclei. White arrowheads indicate CB1R+ cell bodies. SO, stratum oriens; SP, stratum pyramidale; SR, stratum radiatum. (C) The astrocyte marker GFAP (green) shows no overlap with mCherry+ nuclei in the hippocampal CA regions or dentate gyrus (left and middle panels). Inset (middle panel) shows a magnified view of astrocyte nuclei (blue). mCherry+ nuclei occupy the outer third of the dentate gyrus granule cell layer (right panel). ML, molecular layer; GCL, granule cell layer. [file 13064_2021_153_MOESM1_ESM.docx]

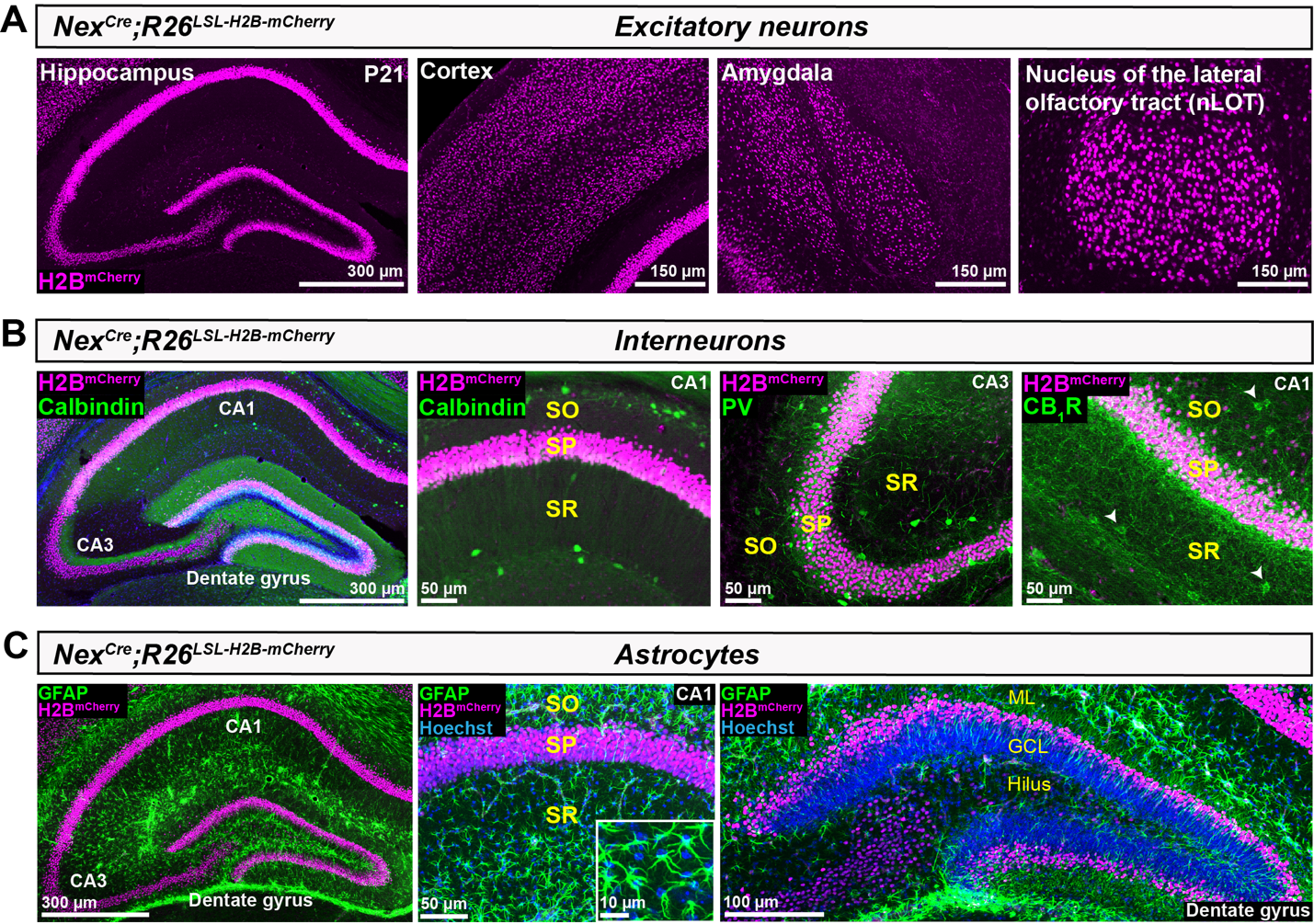


**Figure S1. *Nex^Cre^* drives recombination in forebrain pyramidal neurons but not interneurons or glia**. **(A)** Coronal sections from *Nex^Cre^*;*R26^LSL-H2B-mCherry^* reporter mice at P21 show mCherry+ nuclei (magenta) of pyramidal neurons in the hippocampus, cortex, amygdala, and nucleus of the lateral olfactory tract (nLOT). **(B)** Hippocampal sections from *Nex^Cre^*;*R26^LSL-H2B-mCherry^* reporter mice immunostained for interneuron markers (green) Calbindin (left panels), Parvalbumin (middle panel), and CB_1_R (right­­ panel) show no overlap of interneuron cell bodies with mCherry+ nuclei. White arrowheads indicate CB_1_R+ cell bodies. SO, *stratum oriens*; SP, *stratum pyramidale*; SR, *stratum radiatum*. **(C)** The astrocyte marker GFAP (green) shows no overlap with mCherry+ nuclei in the hippocampal CA regions or dentate gyrus (left and middle panels). Inset (middle panel) shows a magnified view of astrocyte nuclei (blue). mCherry+ nuclei occupy the outer third of the dentate gyrus granule cell layer (right panel). ML, molecular layer; GCL, granule cell layer.
