# Supplementary material for: Neuronal Dystroglycan regulates postnatal development of CCK/cannabinoid receptor-1 interneurons
Source: Neural Dev. 2021 Aug 6;16:4. doi: 10.1186/s13064-021-00153-1 (PMC8349015; doi:10.1186/s13064-021-00153-1)
Supplement: Supplementary file 2 — Additional file 2: Fig. S2. CCK+ interneuron innervation of the dentate gyrus is minimally altered in Dag1cKO mice. (A) Immunostaining of CB1R in the dentate gyrus from P30 Dag1Control (left panels) and Dag1cKO mice (right panels). Single channel images of CB1R (gray) are shown below. (B) Quantification of CB1R pixels for each dentate gyrus layer (*P < 0.05, unpaired two-tailed Student’s t-test; n = 4 mice/genotype). Data are presented as mean values ± s.e.m. Data are normalized to Dag1Control signal in each dentate gyrus layer. OML, outer molecular layer; IML, inner molecular layer; GCL, granule cell layer. [file 13064_2021_153_MOESM2_ESM.docx]

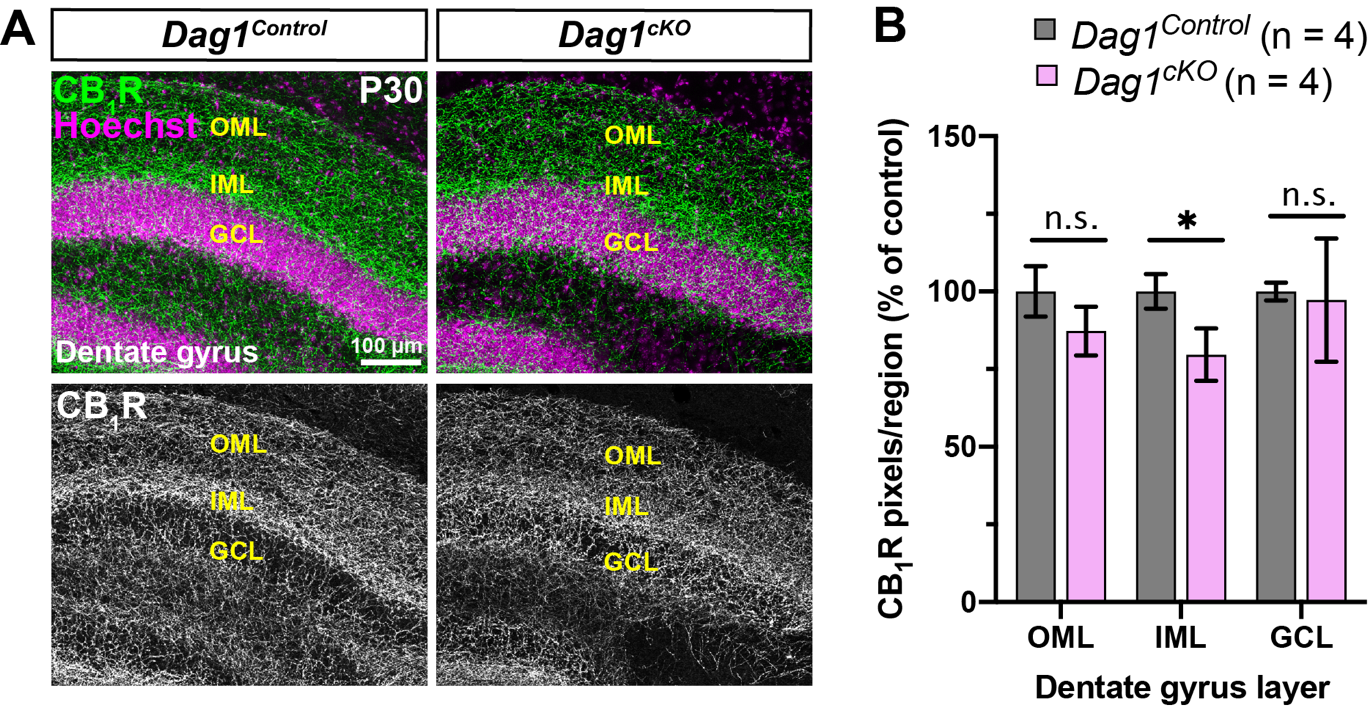


**Figure S2. CCK+ interneuron innervation of the dentate gyrus is minimally altered in *Dag1^cKO^* mice. (A)** Immunostaining of CB_1_R in the dentate gyrus from P30 *Dag1^Control^* (left panels) and *Dag1^cKO^* mice (right panels). Single channel images of CB_1_R (gray) are shown below. **(B)** Quantification of CB_1_R pixels for each dentate gyrus layer (**P* < 0.05, unpaired two-tailed Student’s t-test; n = 4 mice/genotype). Data are presented as mean values ± s.e.m. Data are normalized to *Dag1^Control^* signal in each dentate gyrus layer. OML, outer molecular layer; IML, inner molecular layer; GCL, granule cell layer.
