# Supplementary material for: Neuronal Dystroglycan regulates postnatal development of CCK/cannabinoid receptor-1 interneurons
Source: Neural Dev. 2021 Aug 6;16:4. doi: 10.1186/s13064-021-00153-1 (PMC8349015; doi:10.1186/s13064-021-00153-1)
Supplement: Supplementary file 3 — Additional file 3: Fig. S3. CCK+ interneuron markers are reduced postnatally in Dag1cKO mice. (A) Images of hippocampal CA1 from VGLUT3Cre;Ai9 mice from P3-P18. Immunostaining for tdTomato (green) shows progressive increase in VGLUT3 expression in the pyramidal cell layer (SP, magenta). (B) Immunostaining for VGLUT3 in the CA1 of Dag1Control (top panels) and Dag1cKO mice (bottom panels) from P3-P15. Note the lack of VGLUT3 expression at all ages in Dag1cKO mice. (C) Parvalbumin (PV) labeling is similar in the CA1 of Dag1Control (top panels) and Dag1cKO mice (bottom panels) from P5-P30. [file 13064_2021_153_MOESM3_ESM.docx]

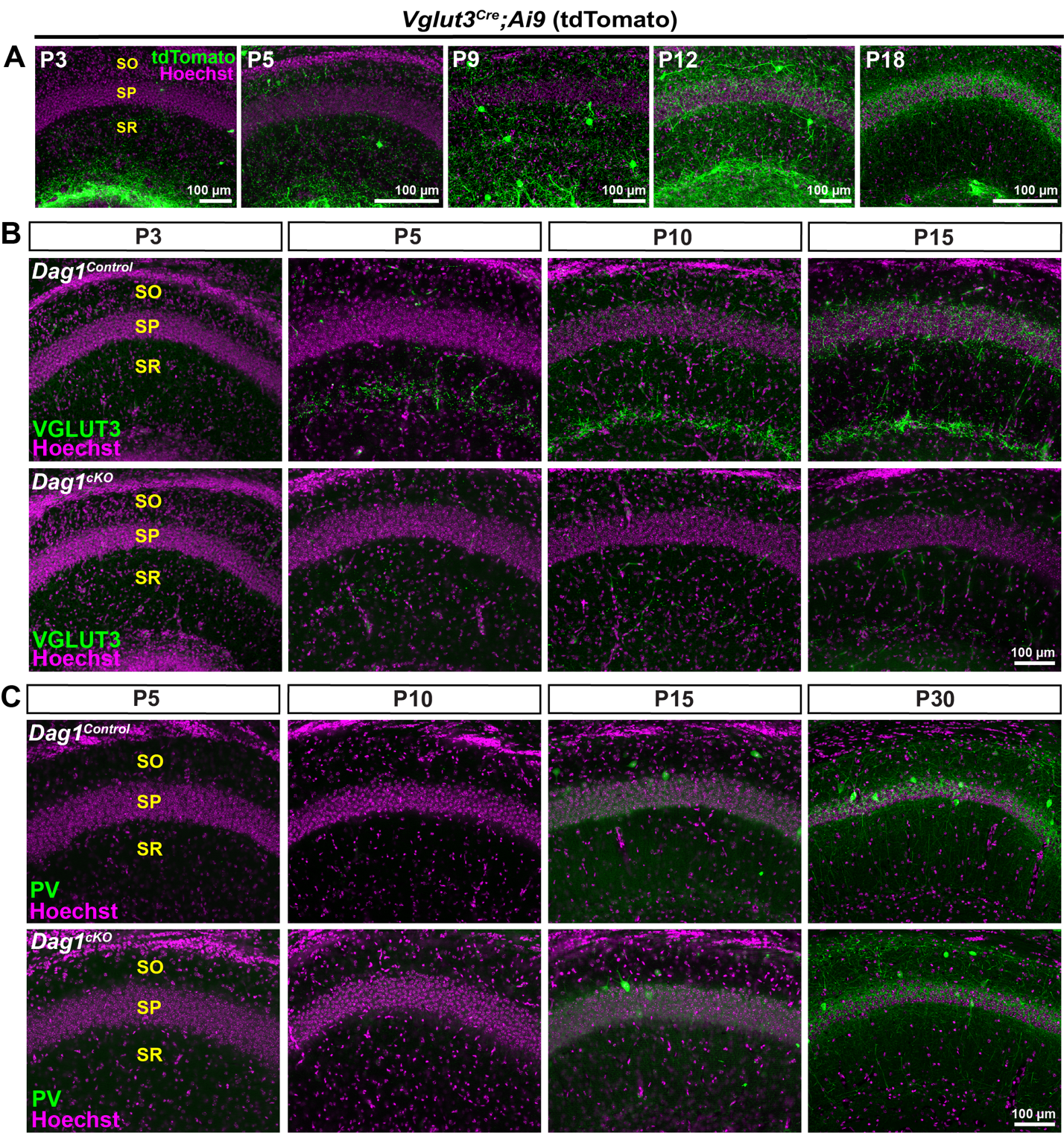


**Figure S3. CCK+ interneuron markers are reduced postnatally in *Dag1^cKO^* mice. (A)** Images of hippocampal CA1 from *VGLUT3^Cre^;Ai9* mice from P3-P18. Immunostaining for tdTomato (green) shows progressive increase in VGLUT3 expression in the pyramidal cell layer (SP, magenta). **(B)** Immunostaining for VGLUT3 in the CA1 of *Dag1^Control^* (top panels) and *Dag1^cKO^* mice (bottom panels) from P3-P15. Note the lack of VGLUT3 expression at all ages in *Dag1^cKO^* mice. **(C)** Parvalbumin (PV) labeling is similar in the CA1 of *Dag1^Control^* (top panels) and *Dag1^cKO^* mice (bottom panels) from P5-P30.
