# Supplementary material for: Neuronal Dystroglycan regulates postnatal development of CCK/cannabinoid receptor-1 interneurons
Source: Neural Dev. 2021 Aug 6;16:4. doi: 10.1186/s13064-021-00153-1 (PMC8349015; doi:10.1186/s13064-021-00153-1)
Supplement: Supplementary file 4 — Additional file 4: Fig. S4. Constitutive deletion of Bax in Dag1cKO mice does not rescue VGLUT3+ terminals. (A-D) Coronal sections of the hippocampus stained for VGLUT3 (gray) from P30 (A) Dag1Control;BaxControl, (B) Dag1Control;BaxKO, (C) Dag1cKO;BaxControl and (D) Dag1cKO;BaxKO mice. (A’-D′) Magnified images of the CA1 (yellow boxed regions) stained for VGLUT3 (green; Right, gray single channel images) and Hoechst (magenta) to stain the pyramidal cell layer (SP). SO, stratum oriens; SP, stratum pyramidale; SR, stratum radiatum. [file 13064_2021_153_MOESM4_ESM.docx]

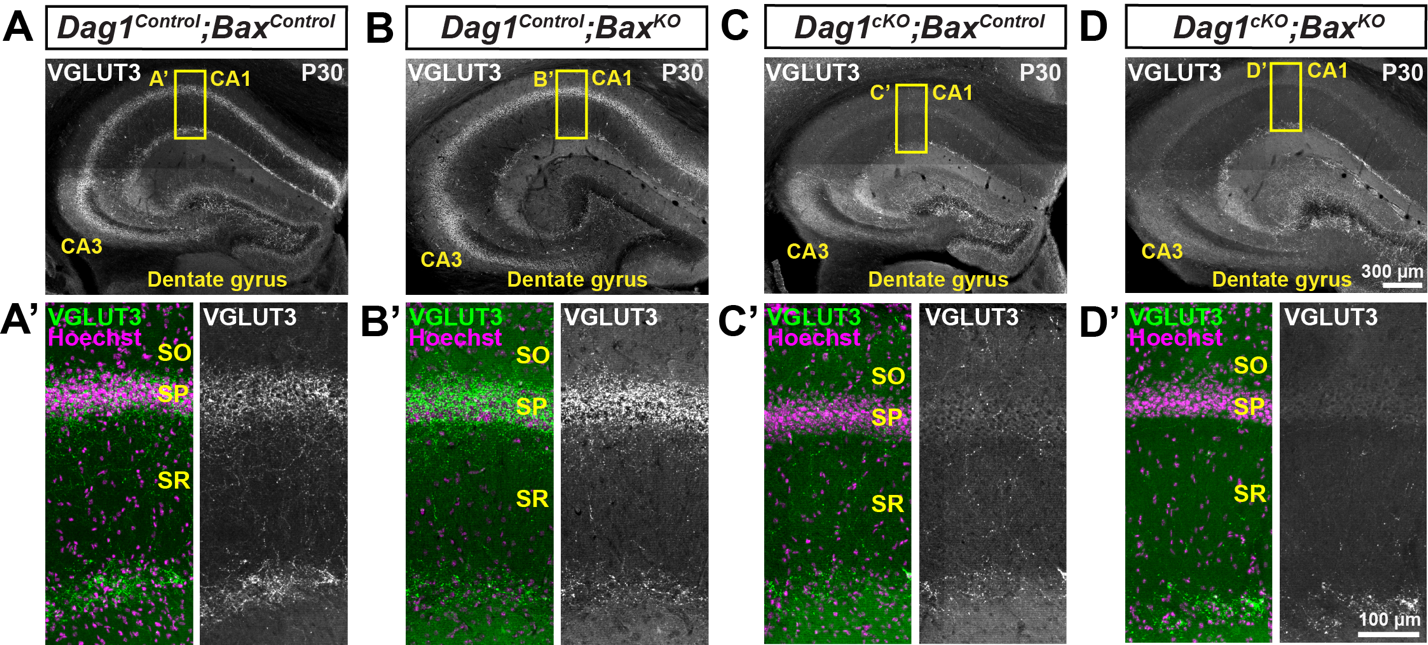


**Figure S4. Constitutive deletion of *Bax* in *Dag1^cKO^* mice does not rescue VGLUT3+ terminals. (A-D)** Coronal sections of the hippocampus stained for VGLUT3 (gray) from P30 **(A)** *Dag1^Control^;Bax^Control^*, **(B)** *Dag1^Control^*;*Bax^KO^*, **(C)** *Dag1^cKO^*;*Bax^Control^* and **(D)** *Dag1^cKO^*;*Bax^KO^* mice. **(A’-D’)** Magnified images of the CA1 (yellow boxed regions) stained for VGLUT3 (green; Right, gray single channel images) and Hoechst (magenta) to stain the pyramidal cell layer (SP). SO, *stratum oriens*; SP, *stratum pyramidale*; SR, *stratum radiatum*.
