# Supplementary material for: Neuronal Dystroglycan regulates postnatal development of CCK/cannabinoid receptor-1 interneurons
Source: Neural Dev. 2021 Aug 6;16:4. doi: 10.1186/s13064-021-00153-1 (PMC8349015; doi:10.1186/s13064-021-00153-1)
Supplement: Supplementary file 5 — Additional file 5: Fig. S5. Constitutive deletion of Bax in Dag1cKO mice does not rescue CB1R+ terminals in the forebrain. (A-C) Coronal sections immunostained for CB1R (green) and Hoechst (magenta) in the cortex (A), amygdala (B), and nucleus of the lateral olfactory tract (C) of P30 Dag1Control;BaxControl, Dag1Control;BaxKO, Dag1cKO;BaxControl and Dag1cKO;BaxKO mice. [file 13064_2021_153_MOESM5_ESM.docx]

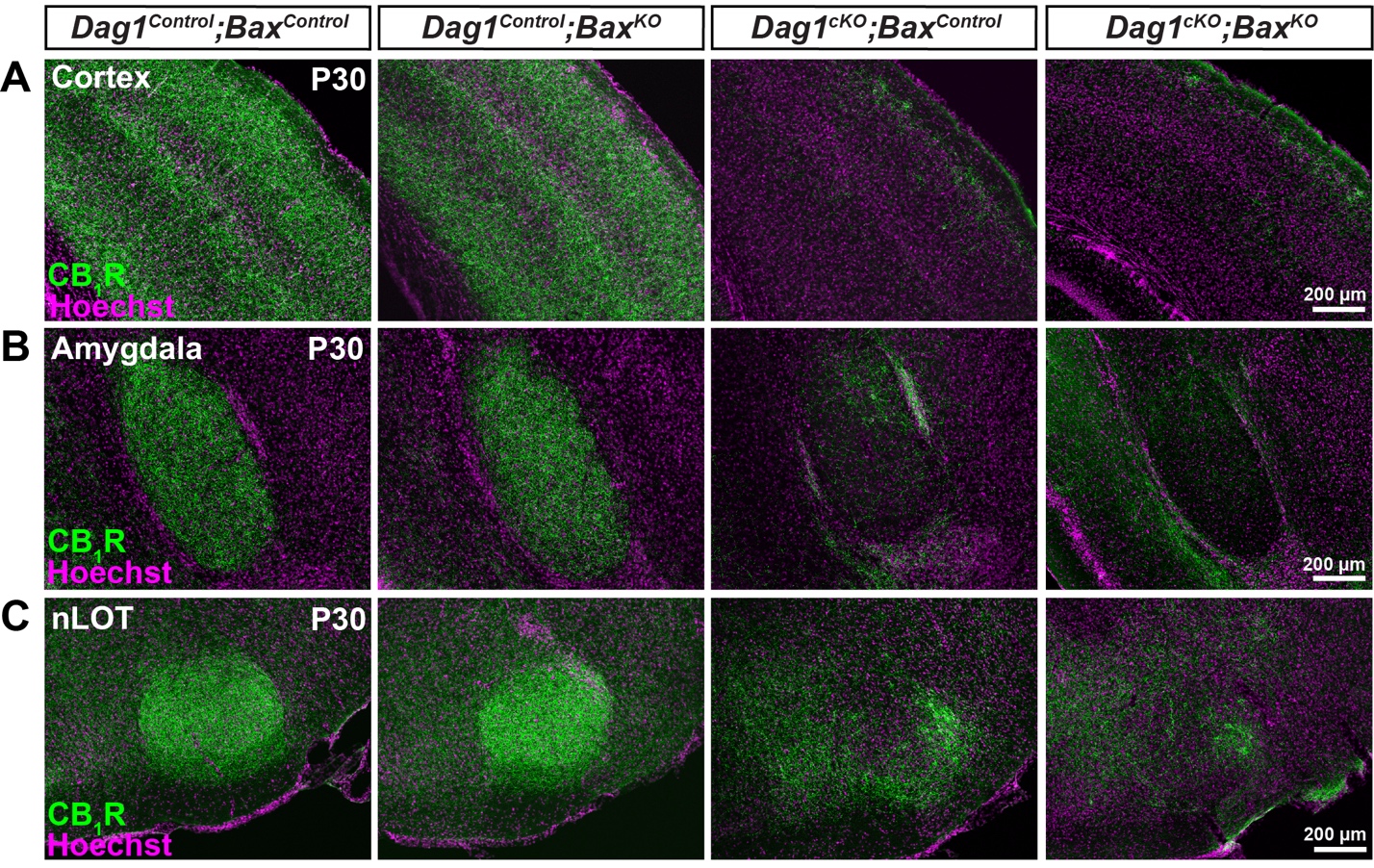


**Figure S5. Constitutive deletion of *Bax* in *Dag1^cKO^* mice does not rescue CB_1_R+ terminals in the forebrain. (A-C)** Coronal sections immunostained for CB_1_R (green) and Hoechst (magenta) in the cortex **(A)**, amygdala **(B)**, and nucleus of the lateral olfactory tract **(C)** of P30 *Dag1^Control^;Bax^Control^*, *Dag1^Control^*;*Bax^KO^*, *Dag1^cKO^*;*Bax^Control^* and *Dag1^cKO^*;*Bax^KO^* mice.
